# The Early Burden of Disability in Individuals With Mood and Other Common Mental Disorders in Ontario, Canada

**DOI:** 10.1001/jamanetworkopen.2020.20213

**Published:** 2020-10-26

**Authors:** Benicio N. Frey, Simone Vigod, Taiane de Azevedo Cardoso, Diego Librenza-Garcia, Lindsay Favotto, Richard Perez, Flavio Kapczinski

**Affiliations:** 1 Mood Disorders Program, Department of Psychiatry and Behavioural Neurosciences, McMaster University, Hamilton, Ontario, Canada; 2 Women’s Health Concerns Clinic, St. Joseph’s Healthcare Hamilton, Hamilton, Ontario, Canada; 3 Women’s College Hospital and Research Institute, Toronto, Ontario, Canada; 4 Department of Psychiatry and Institute for Health Policy, Management and Evaluation, University of Toronto, Toronto, Ontario, Canada; 5 ICES, Toronto, Ontario, Canada; 6 ICES McMaster University, Hamilton, Ontario, Canada

## Abstract

**Importance:**

Large population-based data on the trajectory to disability after the first diagnosis of a mood disorder are lacking.

**Objective:**

To assess the time between an incident mood disorder diagnosis and the receipt of disability services during a follow-up period of as long as 20 years.

**Design, Setting, and Participants:**

This cohort study used health administrative and social service data from ICES for 1 902 792 adults aged 18 to 59 years living in Ontario, Canada. A narrow cohort of individuals who had a new diagnosis of a mood disorder between October 1, 1997, and March 31, 2007, matched by sex and age to individuals with no history of mood disorder, included 278 296 participants. A broader cohort of individuals who had a new diagnosis of other common mental disorders during the same period, matched by sex and age to individuals with no history of mental disorder diagnosis, included 1 624 496 individuals. All individuals were followed up to a maximum end date of March 31, 2017. Data analysis was conducted from November 2017 to June 2018.

**Exposure:**

Incident diagnosis of mood or common mental disorder.

**Main Outcomes and Measures:**

Disability outcomes were as follows: (1) entry into the Ontario Disability Support Program (ODSP), signifying long-term inability to work because of a disability, and (2) admission into a long-term care (LTC) residence, signifying the inability to live in independent housing. Cox proportional hazards models were used.

**Results:**

In the full cohort of 1 902 792 individuals, 278 296 participants (14.6%) were included in the mood disorder cohort (mean [SD] age, 37.5 [11.9] years; 157 386 [56.6%] women), and 1 624 496 participants (85.4%) were included in the common mental disorder cohort (mean [SD], 36.5 [11.8] years; 932 545 [57.4%] women). The incidence of ODSP initiation was greater among individuals with mood disorders than those without (51.5 per 10 000 person-years vs 25.5 per 10 000 person-years; adjusted hazard ratio [aHR], 2.03; 95% CI, 1.95-2.11) and for those with common mental disorders (45.0 per 10 000 person-years vs 27.6 per 10 000 person-years; aHR, 1.57; 95% CI, 1.55-1.60). The aHR for admission to LTC was also higher among individuals with mood disorders compared with those without (aHR, 2.20; 95% CI, 1.80-2.69) and those with common mental disorders compared with those without (aHR, 1.21; 95% CI, 1.14-1.29). Individuals with bipolar disorders had greater ODSP rates than individuals with major depressive disorders (crude rate ratio: 4.31 [95% CI, 3.56-5.17] vs 1.82 [95% CI, 1.36-2.43]).

**Conclusions and Relevance:**

This cohort study found that mood disorders were associated with elevated and early rates of disability services. Effective early intervention strategies targeting functional impairment in this population are encouraged.

## Key Points

**Question:**

What is the risk of receiving disability services after a first (ie, incident) diagnosis of mood or common mental disorder?

**Findings:**

This cohort study included 1 902 792 individuals and found that an incident diagnosis of mood disorder or common mental disorder was associated with high rates of receiving disability services, indicating that mood and common mental disorders are associated with an elevated risk of disability early in the course of illness.

**Meaning:**

The findings of this study suggest that the receipt of disability services occurs early in the trajectory of mood and common mental disorders, indicating that the development of preventative and early intervention strategies focused on functional impairment should be priorities.

## Teaser

This cohort study assesses the time between an incident mood or other common mental disorder diagnosis and the receipt of disability services during a follow-up period of as long as 20 years among adults in Ontario, Canada.

## Introduction

Mood and anxiety disorders are common mental health conditions, affecting 9.5% and 18.1% of the population, respectively.^1^ The Global Burden of Disease Study (2015) showed a large contribution of mental disorders to global disability, with depressive disorders being the third and anxiety disorders being the ninth leading cause of years lived with disability (YLDs).^2^ Overall, mental illnesses accounted for 32.4% of YLDs and 13.0% of disability-adjusted life-years (DALYs) of all disorders.^3^ The cost of mental illnesses is approximately $317 billion per year in the United States^4^ and $50 billion per year in Canada.^4^ These costs include direct costs associated with the health care system as well as the indirect costs related to disability and loss of productivity.

In some jurisdictions, the burden of major depressive disorder in DALYs is greater than the combined burden of breast, colorectal, lung, and prostate cancers.^5^ Occupational costs resulting from absenteeism and presenteeism, medical service costs, and suicide-related costs also contribute to the economic burden associated with major depressive disorder.^6^ Furthermore, major depressive disorder is associated with severe and debilitating chronic medical conditions and is an independent risk factor for cardiovascular morbidity and mortality.^7^ Individuals with bipolar disorder also have a reduced quality of life during both symptomatic (ie, depression or mania) and nonsymptomatic periods, being unable to maintain a proper work role more than 30% of the time.^8,9^ Bipolar disorder more severely affects young people, with long-term consequences for cognition and functioning, being the sixth leading cause of DALYs among people aged 10 to 24 years worldwide.^10^

Although mood disorders are strongly associated with disability, studies in this field are usually limited to specific diagnoses and populations, and there is a lack of large, population-based data.^11^ Small samples and short periods of follow-up limit our knowledge regarding the trajectory of early disability after an incident mood disorder. Understanding the trajectory to disability in high-risk populations, such as individuals with mood disorders, is important given the socioeconomic cost for society and the functional impairment burden for the individuals.^4^ As early intervention strategies can shift the trajectory of mental illnesses toward better long-term health outcomes, characterizing the path to disability among individuals with mood disorders can aid in the development of public health policies that mitigate short-term and long-term disability.

The aim of this study was to compare the risk of receipt of disability services among those with incident mood disorders and other common mental disorders compared with those without during a follow-up period of as long as 20 years. We hypothesized that both individuals with an incident mood disorder (ie, major depressive disorder and bipolar disorder) and incident common mental disorder (ie, anxiety and mood disorders) would have a shorter time to receive disability services compared with individuals matched by age and sex.

## Methods

### Data Sources

Health care in Ontario is government funded, making hospital, emergency, and outpatient care free of charge for all residents. The health administrative data of Ontario residents were accessed at ICES (formerly the Institute for Clinical Evaluative Sciences), in Toronto, Ontario. ICES is an independent, nonprofit research institute funded by an annual grant from the Ontario Ministry of Health and Long-term Care. As a prescribed entity under Ontario’s privacy legislation, ICES is authorized to collect and use health care data for the purposes of health system analysis, evaluation, and decision support, which means that informed consent was not required. Several data sets were accessed for the current study. This study was approved by the Hamilton Integrated Research Ethics Board. This study followed the Strengthening the Reporting of Observational Studies in Epidemiology (STROBE) reporting guideline.

The Registered Persons Database is a population-based registry that contains all health numbers ever issued for the government-funded program and, therefore, provides age, sex, and location of residence for all Ontario residents. Inpatient hospitalizations are captured by the Discharge Abstract Database, which contains administrative information for all admissions to acute care hospitals, and the Ontario Mental Health Reporting System (OMHRS), which contains administrative information for all admissions to adult-designated inpatient mental health beds. The Ontario Health Insurance Plan (OHIP) claims database contains information on inpatient and outpatient services by fee-for-service health care practitioners (primarily physicians) and so-called shadow billings for those paid through non–fee-for-service payment plans. The Ontario Drug Benefit database contains prescription medication claims for those covered under the Ontario Disability Support Program (ODSP). This includes individuals aged 65 years and older and recipients of social assistance. Data on immigration status were provided by the Immigration, Refugees, and Citizenship Canada Permanent Resident Database. These data sets were linked using unique encoded identifiers and analyzed at ICES.

### Sample

For the exposed groups, we considered all Ontario residents aged 18 to 59 years with an incident (ie, new or first-onset) mood disorder diagnosis between October 1, 1997, and March 31, 2017, either on hospital discharge, using the Discharge Abstract Database or the OMHRS, or during an outpatient visit, using records from the OHIP database. Two groups were studied. The first (ie, the mood disorder cohort) included individuals with at least 1 outpatient visit with OHIP code 296 or 311 or an *International Classification of Diseases, Ninth Revision *(*ICD-9*) code for bipolar or major depressive disorder or at least 1 hospitalization with an *ICD*-*9* code for bipolar or major depressive disorder (before 2002); *International Statistical Classification of Diseases and Related Health Problems, Tenth Revision *(*ICD-10*) codes F30-34, F38, or F39; or OMHRS *Diagnostic and Statistical Manual of Mental Disorders* (Fourth Edition) diagnoses of bipolar or major depressive disorder. All specific diagnoses are detailed in eTable 1 in the Supplement. The second group (ie, the common mental disorder cohort) included individuals from the mood disorder group as well as individuals with at least 2 OHIP outpatient codes 300 within 1 year or at least 1 hospitalization with *ICD-9* diagnoses of anxiety, somatoform, and related disorders (before 2002); *ICD*-*10* codes F53.0, F40 to F42, F44, F45.0, F45.2, F48, or F68.0; or OMHRS DSM diagnoses of anxiety, somatoform, and related disorders (specific diagnoses are detailed in eTable 1 in the Supplement). This broader set of codes was intended to capture individuals with common mental disorders. We required 2 codes within 1 year or a single 300 code and any other code within 1 year for inclusion in this group to avoid excessively compromising specificity. The first point of contact during the accrual period was selected as the index event. More detailed information regarding the coding procedures is available in eTable 1 in the Supplement. Individuals were excluded if (1) their records did not link with administrative records (invalid key number, death date prior to index date); (2) the patient did not access any health care service in the 7 years before their incident mood disorder diagnosis; (3) the patient was not eligible for the OHIP at the time of incident mood disorder diagnosis or during the 5-year look-back window; (4) the patient was already a resident of a long-term care (LTC) facility on the date of the incident mood disorder diagnosis or 6 months prior; (5) the patient already had an ODSP claim up to 6 months prior to the date of their incident mood disorder diagnosis; or (6) the patient had any other mental health–related or addiction-related outpatient or inpatient claim 5 years before their incident mood disorder diagnosis (eTable 2 in the Supplement). Each individual with a diagnosis was matched at a 1:1 ratio based on year of birth and sex to an Ontario resident who had no hospitalization or physician visit for mental illness or addiction during the accrual period. Individuals were followed up until disability, death, loss of OHIP eligibility, or completing age 65 years, with a final follow-up date of March 31, 2017. Individuals in the unexposed groups were censored if they used health care services (inpatient or outpatient) for a mental illness or addiction during the follow up period.

### Outcomes

#### ODSP

Initiation of ODSP benefits was determined using the Ontario Drug Benefit (ODB) claims data set. The date of the first claim that listed ODSP as the method of coverage was used as the date of ODSP initiation. Only claims for individuals younger than 65 years were considered because all individuals aged 65 years or older receive coverage in general, which precludes the need for ODSP. In Ontario, to qualify for disability support under the ODSP, individuals must be aged at least 18 years, be an Ontario resident, be in financial need, and meet the program’s definition of a person with a disability or be a member of a prescribed class. ODBP’s definition of a person with a disability means that they have a substantial mental or physical impairment that is continuous or recurrent and that is expected to last more than 1 year, causing significant impairment in the individual’s ability to work, to perform self-care, or to take part in community life. The impairment, its duration, and restrictions must have been verified by an approved health care professional.^12^

#### LTC Residence

Admission into an LTC home was identified based on 2 records of an ODB claim from an LTC home or an OHIP record with a fee code indicating that the service was completed at an LTC home within 90 days. The first claim was used as the date of LTC entrance. To be eligible for admission to LTC, individuals must be aged 18 years or older, have a valid OHIP card, and have care needs, including 24-hour nursing care and personal care, frequent assistance with activities of daily living, onsite supervision or monitoring to ensure safety or well-being, needs that cannot be safely met in the community through publicly funded community-based services and other caregiving support, and needs that can be met in an LTC home.^13^

### Covariates

Sociodemographic variables at the time of index mood disorder diagnoses included age, sex, rurality, community size, neighborhood income quintile, immigration status, chronic conditions, comorbidities, and level of social marginalization. Comorbidities were assessed with the Charlson Comorbidity Index score, which was calculated using a 5-year look-back, prior to the mood disorder diagnosis. Rurality was assessed with the Rurality Index of Ontario, and social marginalization was assessed with the Ontario Marginalization Index. More details regarding these instruments can be found in eTable 3 and eTable 4 in the Supplement.

### Statistical Analysis

Frequency and proportions of baseline demographic variables were calculated for both the mood disorder and the common mental disorder groups and the matched individuals with no mental disorder diagnosis. The proportion of each cohort that received disability services (ODSP or LTC) for each exposed group and for the matched unexposed group was calculated, along with the total person-time (actual time-at-risk) that all patients contributed to the study. Crude rates of disability services among the exposed and unexposed groups were calculated by dividing the frequency of receipt of disability services in each cohort by the total person-time. Crude rate ratios were also calculated ([crude rate of receipt of disability services of exposed group / crude rate of receipt of disability services of unexposed] × 10 000 person-years) for the overall cohort and by age and sex groups. Receipt of disability services and death after an incident mental health disorder diagnosis was described using the cumulative incidence function for a period of as long as 20 years. Time to disability was analyzed using the Fine and Gray competing risk subdistribution hazard model,^14^ considering death as a competing risk. Individuals with no psychiatric disorder diagnosis at index date were used as comparators in the unexposed group. Models were adjusted for rurality, income quintile, Charlson Comorbidity Index score, and year of diagnosis. Additional analyses were conducted separately, stratifying the mood disorder cohort into major depressive and bipolar disorders. Crude rate of disability, crude rate ratios, and adjusted hazard ratios were recalculated for the stratified cohorts. The codes used to define major depressive and bipolar disorders are detailed in eTable 1 in the Supplement. Data analysis was conducted in SAS Enterprise Guide version 7.1 (SAS Institute). Statistical significance was set at *P* < .05, and all tests were 2-tailed.

## Results

### Participants

**Figure 1.**
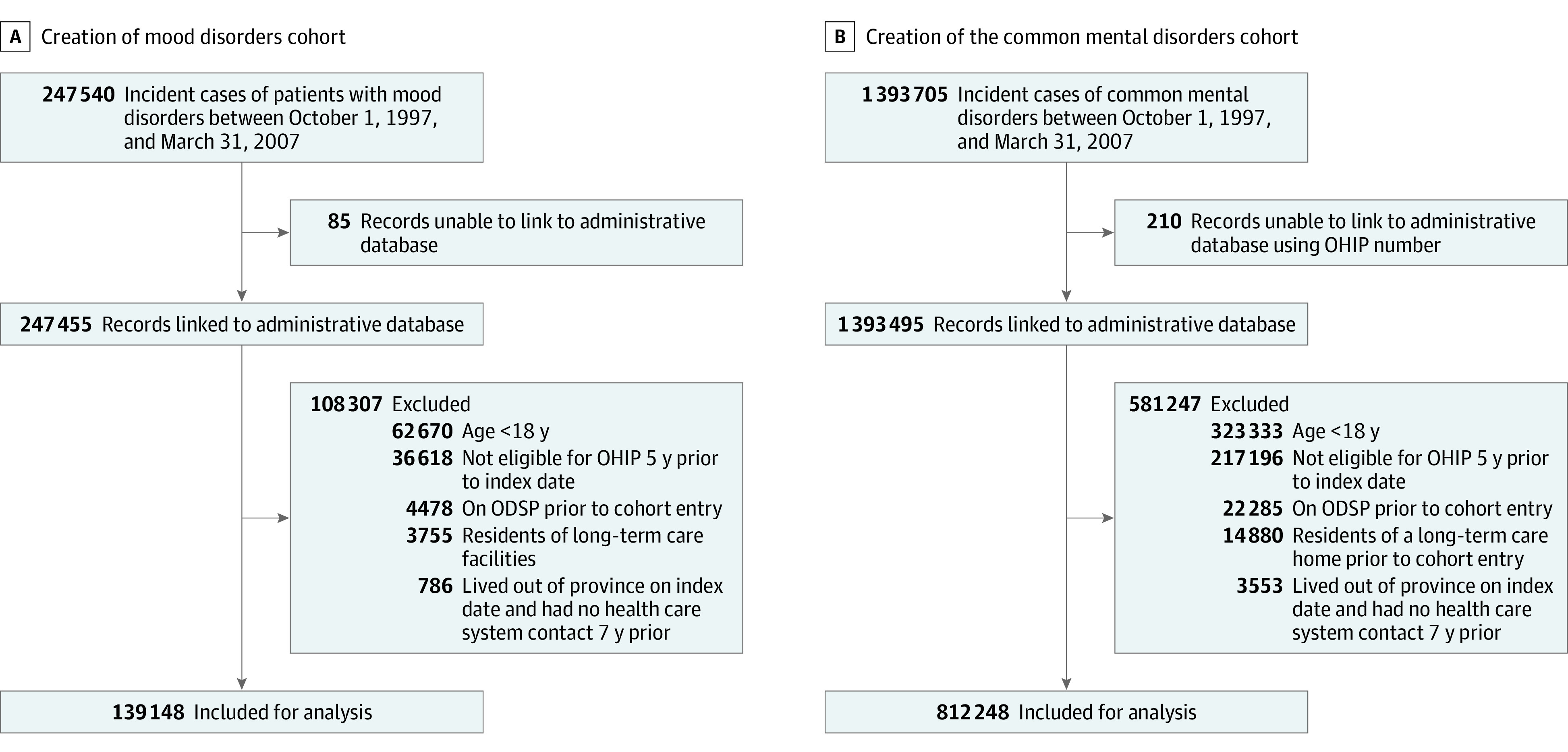
Creation of the 2 Cohorts ODSP indicates Ontario Disability Support Program; and OHIP, Ontario Health Insurance Plan.

After assessment for the inclusion criteria, we included a total of 1 902 792 individuals: 278 296 (14.6%) in the mood disorder cohort, with 139 148 individuals (50.0%) with a mood disorder diagnosis and 139 148 matched individuals (50.0%) with no mood disorder diagnosis, and 1 624 496 (85.4%) in the common mental disorder cohort, with 812 248 individuals (50.0%) with a common mental disorder diagnosis and 812 248 matched individuals (50.0%) with no common mental disorder diagnosis (Figure 1).

### Descriptive Data

Sociodemographic characteristics of participants appear in Table 1 and eTable 3 in the Supplement. The mean (SD) age was 37.5 (11.9) years for the mood disorder cohort and 36.5 (11.8) years for the common mental disorder cohort. Of 278 296 individuals with and without mood disorders in the mood disorder cohort, 157 386 (56.6%) were women. Of 1 624 496 individuals with and without common mental disorders in the common mental disorder cohort, 932 545 (57.4%) were women. Most participants came into the exposed group via outpatient contact (Table 1), and the most common clinical comorbidities were asthma, hypertension, and diabetes (eTable 3 in the Supplement).

**Table 1.** Baseline Sociodemographic Information for Individuals With Mood Disorders, Common Mental Disorders, and Matched Individuals

Characteristic	Patients, No. (%)			
	With mood disorder (n = 139 148)	Without mood disorder (n = 139 148)	With common mental disorder (n = 812 248)	Without common mental disorder (n = 812 248)
Age, mean (SD), y	37.54 (11.94)	37.54 (11.96)	37.72 (11.79)	37.72 (11.80)
Women	78 691 (56.55)	78 695 (56.55)	466 273 (57.41)	466 272 (57.41)
Died	4911 (3.53)	1830 (1.32)	26 957 (3.32)	19 084 (2.35)
Incident event type				
Inpatient	1080 (0.78)	NA	1325 (0.16)	NA
Outpatient	138 068 (99.22)	NA	810 923 (99.84)	NA
Immigrant status				
<10 y	5567 (4.00)	7945 (5.71)	36 582 (4.50)	48 778 (6.01)
≥10 y	5912 (4.25)	8166 (5.87)	37 826 (4.66)	45 898 (5.65)
Nonimmigrant	127 669 (91.75)	123 037 (88.42)	737 840 (90.84)	717 572 (88.34)

Abbreviation: NA, not applicable.

### Outcome Data

eTable 5 in the Supplement shows the proportion of receipt of disability services (ie, ODSP) and LTC between individuals with and without mood disorders. The proportion and crude rate of individuals with a mood disorder who were approved to receive ODSP was greater overall at all age groups (eTable 5 in the Supplement). The incidence of ODSP initiation was greater among individuals with mood disorders than those without (51.5 per 10 000 person-years vs 25.5 per 10 000 person-years). The crude rate ratio of ODSP for individuals with a mood disorder vs those without ranged from 1.06 (95% CI, 0.93-1.18) for women aged 51 to 60 years and 4.13 (95% CI, 3.68-4.53) for men aged 18 to 30 years (Table 2). In addition, the proportion and crude rate of individuals with mood disorders who transitioned to LTC compared with individuals with no mood disorder was greater overall and at all age groups, except for women aged 18 to 30 years (eTable 5 in the Supplement). The crude rate ratio of LTC for individuals with a mood disorder vs those without ranged between 0.82 (95% CI, 0.15-0.94) for women aged 18 to 30 years and 4.84 (95% CI, 2.37-6.05) for men aged 41 to 50 years (Table 2).

**Table 2.** Crude Rate Ratio of Disability of Individuals With Mood Disorder and Common Mental Disorders Compared With Matched Individuals

Characteristic	ODSP	LTC
	Crude rate ratio (95% CI)	Crude rate ratio (95% CI)
**Mood disorder cohort, 139 148 individuals with mood disorder**		
Overall	2.02 (1.94-2.10)	2.60 (2.08-3.02)
Women		
Aged 18-30 y	1.12 (1.00-1.23)	0.82 (0.15-0.94)
Aged 31-40 y	1.86 (1.65-2.05)	1.44 (0.42-1.74)
Aged 41-50 y	1.63 (1.45-1.79)	2.47 (1.36-3.09)
Aged 51-60 y	1.06 (0.93-1.18)	1.99 (1.20-2.47)
Men		
Aged 18-30 y	4.13 (3.68-4.53)	1.23 (0.22-1.41)
Aged 31-40 y	2.55 (2.25-2.83)	2.99 (0.52-3.42)
Aged 41-50 y	2.45 (2.16-2.70)	4.84 (2.37-6.05)
Aged 51-60 y	1.27 (1.11-1.42)	3.81 (2.01-4.76)
**Common mental disorder cohort, 812 248 individuals with common mental disorders**		
Overall	1.63 (1.61-1.66)	1.30 (1.22-1.38)
Women		
Aged 18-30 y	1.48 (1.41-1.55)	1.12 (0.49-1.39)
Aged 31-40 y	1.50 (1.44-1.56)	1.01 (0.78-1.18)
Aged 41-50 y	1.32 (1.27-1.37)	1.15 (1.00-1.28)
Aged 51-60 y	0.93 (0.88-0.97)	1.30 (1.12-1.46)
Men		
Aged 18-30 y	3.42 (3.19-3.64)	3.12 (1.31-3.88)
Aged 31-40 y	2.07 (1.98-2.15)	1.46 (1.13-1.71)
Aged 41-50 y	1.55 (1.49-1.61)	1.46 (1.25-1.64)
Aged 51-60 y	1.20 (1.14-1.26)	1.44 (1.23-1.61)

Abbreviations: LTC, long-term care; ODSP, Ontario Disability Support Program.

eTable 6 in the Supplement presents the proportion and crude rate of the individuals in the common mental disorder cohort who transitioned to disability services (ODSP and LTC). The rate was greater overall at all at age groups for individuals with common mental disorders compared with individuals with no common mental disorders. The overall crude rate of receiving ODSP was 45.0 per 10 000 person-years for the exposed group and 27.6 per 10 000 person-years for matched individuals in the unexposed group. The overall crude rate of transitioning to LTC was 2.23 per 10 000 person-years for the exposed group and 1.71 per 10 000 person-years for the unexposed group. The crude rate ratio of ODSP for individuals with common mental disorders compared with those without ranged between 0.93 (95% CI, 0.88-0.97) for women aged 51 to 60 years and 3.42 (95% CI, 3.19-3.64) for men aged 18 to 30 years (Table 2). Notably, the overall crude rate ratio of ODSP was greater for the mood disorder cohort (2.02; 95% CI, 1.94-2.10) than for the common mental disorder cohort (1.63; 95% CI, 1.61-1.66) (Table 2). The crude rate ratio of LTC for the common mental disorders cohort ranged between 1.01 (95% CI, 0.78-1.18) for women aged 31 to 40 years and 3.12 (95% CI, 1.31-3.88) for men aged 18 to 30 years (Table 2). Similar to the observed rates of ODSP, the overall crude rate ratio of LTC was also greater for the mood disorder cohort (2.60; 95% CI, 2.08-3.02) than the common mental disorder cohort (1.30; 95% CI, 1.22-1.38) (Table 2).

**Figure 2.**
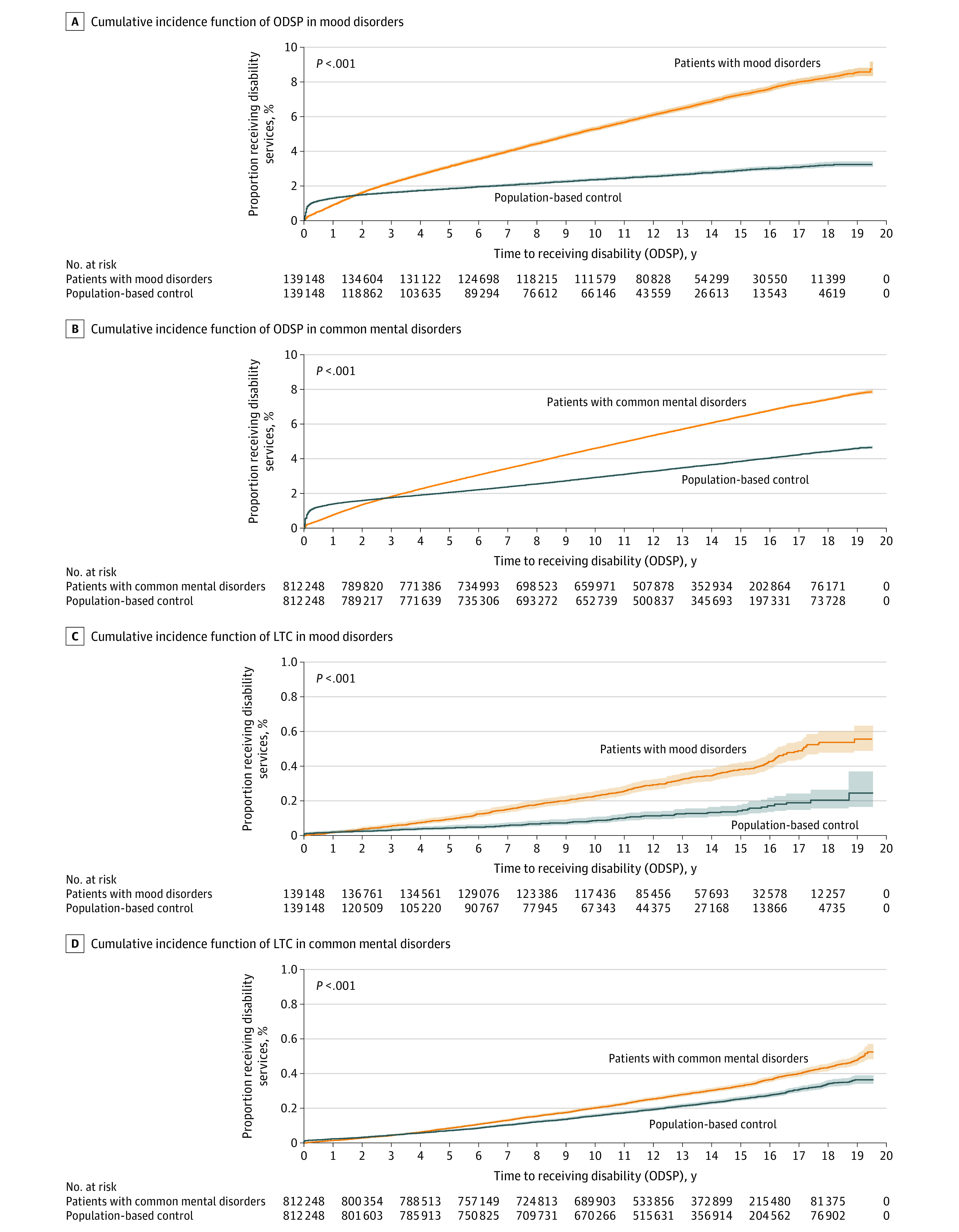
Cumulative Incidence Function of Ontario Disability Support Program (ODSP) and Long-term Care (LTC) in Mood Disorder Cohort and Common Mental Disorder Cohort Individuals with mood disorders were compared with age-matched and sex-matched individuals with no mood disorder (139 148 individuals per group). Individuals with common mental disorders were compared with age-matched and sex-matched individuals with no common mental disorder (812 248 individuals per group). Shaded areas indicate 95% CIs.

Figure 2A and Figure 2B depict the cumulative incidence function of acceptance to ODSP among individuals with mood disorders and common mental disorders. There was a sharper incidence of acceptance to ODSP in individuals with mood and common mental disorders shortly after the incident psychiatric diagnosis, with a steeper cumulative incidence during the 20 years of follow up.

The hazard rate of ODSP was 2.03 (95% CI, 1.95-2.11) times greater for individuals with mood disorders vs those without and 1.57 (95% CI, 1.55-1.60) times greater for individuals with common mental disorders vs those without, after adjusting for income quintile, rurality, chronic conditions, and year of incident diagnosis (Table 3). Both age at time of diagnosis and sex significantly modified the rate of acceptance to ODSP. The hazard ratio of ODSP in patients with mood disorders and common mental disorders was greater in younger men.

**Table 3.** Time to Disability Among Patients With Mood Disorders and Common Mental Disorders Compared With Matched Individuals

Characteristic	ODSP		LTC	
	aHR^a^	*P* value	aHR^a^	*P* value
**Mood disorder cohort, 139 148 individuals with mood disorder**				
Overall^b^	2.03 (1.95-2.11)	<.001	2.20 (1.80-2.69)	<.001
Women				
Aged 18-30 y	2.00 (1.80-2.22)	<.001	0.88 (0.30-2.58)	.81
Aged 31-40 y	1.96 (1.75-2.19)	<.001	1.19 (0.58-2.44)	.64
Aged 41-50 y	1.68 (1.50-1.88)	<.001	2.04 (1.29-3.25)	.003
Aged 51-60 y	1.11 (0.98-1.26)	.10	1.77 (1.20-2.61)	.004
Men				
Aged 18-30 y	4.22 (3.72-4.78)	<.001	2.56 (0.69-9.48)	.16
Aged 31-40 y	2.48 (2.19-2.81)	<.001	2.33 (0.95-5.71)	.06
Aged 41-50 y	2.34 (2.08-2.64)	<.001	3.76 (2.25-6.29)	<.001
Aged 51-60 y	1.22 (1.06-1.39)	.005	3.16 (1.95-5.13)	<.001
**Common mental disorder cohort, 812 248 individuals with common mental disorders**				
Overall^b^	1.57 (1.55-1.60)	<.001	1.21 (1.14-1.29)	<.001
Women				
Aged 18-30 y	1.63 (1.57-1.69)	<.001	0.97 (0.70-1.33)	.85
Aged 31-40 y	1.49 (1.43-1.55)	<.001	0.97 (0.78-1.22)	.81
Aged 41-50 y	1.28 (1.23-1.33)	<.001	1.08 (0.94-1.23)	.28
Aged 51-60 y	0.88 (0.84-0.93)	<.001	1.21 (1.06-1.40)	.01
Men				
Aged 18-30 y	2.89 (2.76-3.01)	<.001	2.45 (1.68-3.57)	<.001
Aged 31-40 y	1.99 (1.90-2.08)	<.001	1.37 (1.09-1.73)	.01
Aged 41-50 y	1.49 (1.43-1.56)	<.001	1.34 (1.16-1.55)	<.001
Aged 51-60 y	1.12 (1.06-1.18)	.001	1.26 (1.09-1.46)	.002

Abbreviations: aHR, adjusted hazard ratio; LTC, long-term care; ODSP, Ontario Disability Support Program.

^a^ Adjusted for neighborhood income quintile, rurality, Charlson Comorbidity Index score, and year of incident diagnosis.

^b^ Models were stratified on age and sex after identifying significant interaction (ie, *P* < .001) for age and sex.

Figure 2C and Figure 2D depict the cumulative incidence function of LTC among patients with mood disorders and common mental disorders. There was a steady steeper cumulative incidence of LTC in individuals with mood and common mental disorders during the 20 years of follow up compared with those with no diagnosis.

Finally, the hazard rate of LTC was 2.20 (95% CI, 1.80-2.69) times greater for patients with mood disorders and 1.21 (95% CI, 1.14-1.29) times greater for patients with common mental disorders than the rate of LTC for patients in the unexposed groups, after adjusting for income quintile, rurality, chronic conditions, and year of incident diagnosis. Sex significantly modified the rate of transition to LTC. The hazard ratio of LTC in both mood disorder and common mental disorder patients was greater in men across all ages.

Additional analyses stratifying the mood disorders group into individuals with bipolar disorders (n = 10 981) and major depressive disorders (n = 128 167) (eTable 7 in the Supplement) suggest greater rates of ODSP among individuals with bipolar disorders (crude rate ratio: 4.31 [95% CI, 3.56-5.17] vs 1.82 [95% CI, 1.36-2.43]) (eTables 8-12, eFigure 1, and eFigure 2 in the Supplement). While results suggest a similar trend for transition to LTC (crude rate ratio: 5.25 [95% CI, 1.88-11.58] vs 2.34 [95% CI, 0.35-7.82]), wide confidence intervals do not support a definite conclusion of differences between the 2 mood disorder groups (eTables 8-12, eFigure 3, and eFigure 4 in the Supplement).

## Discussion

In a large population-based cohort study, we found that an incident diagnosis of mood disorder or common mental disorder was associated with high rates of receipt of disability services. The rates of disability services were greater in individuals with incident mood disorders than those who received a diagnosis of common mental disorders. These results indicate that mood and common mental disorders are associated with an elevated risk of receipt of disability services early in the course of illness.

Notably, mood disorders most typically start during adolescence or early adulthood, with a significant negative impact on educational achievement and stable employment. Our findings are consistent with the Global Burden of Disease Study 2010, which reported that mental and behavioral disorders are a major cause of YLDs, contributing to 36% of all 289 diseases in individuals aged 20 to 29 years.^15^ Similarly, a community-based study in the United States with 4501 individuals found that those with mood and anxiety disorders had greater odds of being disabled or temporarily laid off and looking for a job after 10 years of follow-up.^16^ Longitudinal studies conducted in primary care^17^ and specialized clinical services^18^ found that 1 in 5 individuals with depressive disorders are granted disability pension in 5 years of follow-up. Notably, common mental disorders, such as depression and anxiety, also increase the likelihood of subsequent disability pension for non–mental health conditions, and this association is stronger in younger people than older adults.^19^ Prospective studies also showed that individuals with bipolar disorder with occupational disability at baseline had poorer treatment response, more illness recurrences, and worse functioning and quality of life after 6 to 12 months of follow-up.^20,21^ An inability to achieve financial independence has been associated with social isolation and/or marginalization, loss of purpose, and low self-esteem, which further negatively affect mental health, creating a vicious cycle that might “trap the individual in inactivity and poverty.”^22^ Many factors have been associated with mental health disorders and work disability. Among these, probably the main factor associated with work disability has been the severity of depressive symptoms over time.^17,20,23,24^ However, although symptom improvement is associated with overall functional improvement, functional impairment is frequently observed even after symptom recovery. It has been suggested that cognitive deficits observed in individuals with mental disorders during clinical remission of symptoms account for some of the functional and occupational impairment in this population.^11^ Another main factor associated with work disability in individuals with common mental disorders is the presence of comorbid physical illness. In addition, large cross-sectional and longitudinal studies have found that disability associated with common mental disorders are comparable or greater than those associated with highly disabling physical illnesses, such as back pain, arthritis, and heart disease.^24,25^

Another major finding of this study is that men had a greater hazard ratio for receiving disability services than women. This result is consistent with previous studies showing that male sex was associated with greater work disability.^17^ While the reasons why men have an earlier path to disability services than women are unknown, a previous longitudinal study^26^ found that the worse psychosocial functioning observed in boys with depressive and anxiety symptoms was associated with lower self-esteem, poorer academic performance, behavioral problems, and social isolation. Therefore, the early consequences of mental illness seems to be greater in boys and men than girls and women in part owing to a greater negative association with self-esteem, loss of social support, and poor coping. Development of effective treatment strategies targeting these domains early, shortly after the onset of mood and other common mental disorders, are encouraged.

Results from this study have relevant health care and health policy implications. From a health care perspective, treatment planning following symptomatic recovery of an incident common mental disorder should include assessment and/or management of comorbid physical conditions, psychoeducation, self-esteem, social and cognitive functioning, and enhancement of coping. Recently, treatment planning based on staging models have been advocated for common mental health disorders.^27^ For instance, there is evidence that structured psychoeducation and cognitive behavioral therapy, 2 widely used first-line interventions for mood disorders, are more effective earlier in the course of mood disorders,^28,29^ although these types of treatment will likely need to be adapted for younger populations.^30^ Monitoring of suicidal behavioral is also critical in younger populations, given that receipt of disability pension has been shown to increase the risk of suicide 4-fold in a large sample of adults with mental disorders aged 19 to 23 years.^22^ Considering the extremely high costs of common mental disorders worldwide^31,32^ and the fact that indirect costs associated with job loss and disability exceed the direct costs of treatment, our results are also relevant for health policy. Future research on the impact of the integration of health care and work policies on common mental disorders is warranted.

### Limitations

This study has limitations. Limitations are those common to the use of health care administrative databases and may include misclassification of exposure group status because only individuals with physician and hospital-based health service utilization for mental disorders were included in the exposed group. This could lead to underestimation of the effect size if individuals with mental disorders who were not in contact with the health care system were in the unexposed group. It also must be acknowledged that transition to the public health care and social disability benefits system and/or an LTC facility are highly specific outcomes in relation to disability; these data sets do not capture other more subtle or patient-reported aspects of disability, again suggesting that the full association of mood disorders with disability status might be underestimated here. In addition, because the data we used were collected for health administrative purposes and not planned as variables of interest to be collected for the current study, we were not able to measure all potential confounders or explanatory factors in the association between mood disorder and receipt of disability services. These might include symptom severity, medication adherence or nonadherence, or treatment response as well as familial, social, and vocational support opportunities. Such variables would be important to explore in future clinical research in this area to guide intervention. Other limitations include the relatively low absolute rates of receipt of ODSP and LTC and the fact that we did not have access to the primary diagnosis leading to approval for ODSP or LTC. Therefore, we could not ascertain whether the elevated rates of ODSP and LTC in individuals with mood disorders were primarily associated with a mental or a physical condition. Notwithstanding these limitations, the fact that individuals with mood disorders received disability services earlier than those with no mood disorder (regardless of the specific diagnosis used for approval for disability services) is novel and sends a strong message to health care services and policy makers regarding the early burden associated with mood disorders.

## Conclusions

In this cohort study, an incident diagnosis of mood and common mental disorders was associated with higher rates of receipt of disability services. This association was greater in men and in individuals with a specific diagnosis of mood disorder. These results suggest a path to disability services early in the course of common mental disorders. Integration of work and health care strategies to mitigate such risk are encouraged.

## Notes

Supplement. **eTable 1. **Diagnosis Codes **eTable 2. **Selection of Cohorts **eTable 3. **Complete Sociodemographic Information for Cohorts of Individuals With Mood Disorders, Common Mental Disorders, and Matched Individuals **eTable 4. **Algorithms Used to Identify Chronic Conditions at Time of Mood Disorder Diagnosis **eTable 5. **Crude Rates of Disability for the Mood Disorder Cohort **eTable 6. **Crude Rates of Disability for the Common Mental Disorder Cohort **eTable 7. **Baseline Sociodemographic Information for Individuals with Major Depressive Disorders, Bipolar Disorders, and Matched Individuals **eTable 8. **Crude Rates of Disability for Bipolar Disorder **eTable 9. **Crude Rates of Disability for Major Depressive Disorder **eTable 10. **Crude Rate Ratio of Disability for Bipolar Disorder **eTable 11. **Crude Rate Ratio of Disability for Major Depressive Disorder **eTable 12. **Adjusted Hazard Ratios for Major Depressive and Bipolar Disorders **eFigure 1. **Cumulative Incidence Function of ODSP in Bipolar Disorder **eFigure 2. **Cumulative Incidence Function of ODSP in Major Depressive Disorder **eFigure 3. **Cumulative Incidence Function of LTC in Bipolar Disorder **eFigure 4. **Cumulative Incidence Function of LTC in Major Depressive Disorder
