# Supplementary material for: The Early Burden of Disability in Individuals With Mood and Other Common Mental Disorders in Ontario, Canada
Source: JAMA Netw Open. 2020 Oct 26;3(10):e2020213. doi: 10.1001/jamanetworkopen.2020.20213 (PMC7588941; doi:10.1001/jamanetworkopen.2020.20213)
Supplement: Supplement. — eTable 1. Diagnosis Codes eTable 2. Selection of Cohorts eTable 3. Complete Sociodemographic Information for Cohorts of Individuals With Mood Disorders, Common Mental Disorders, and Matched Individuals eTable 4. Algorithms Used to Identify Chronic Conditions at Time of Mood Disorder Diagnosis eTable 5. Crude Rates of Disability for the Mood Disorder Cohort eTable 6. Crude Rates of Disability for the Common Mental Disorder Cohort eTable 7. Baseline Sociodemographic Information for Individuals with Major Depressive Disorders, Bipolar Disorders, and Matched Individuals eTable 8. Crude Rates of Disability for Bipolar Disorder eTable 9. Crude Rates of Disability for Major Depressive Disorder eTable 10. Crude Rate Ratio of Disability for Bipolar Disorder eTable 11. Crude Rate Ratio of Disability for Major Depressive Disorder eTable 12. Adjusted Hazard Ratios for Major Depressive and Bipolar Disorders eFigure 1. Cumulative Incidence Function of ODSP in Bipolar Disorder eFigure 2. Cumulative Incidence Function of ODSP in Major Depressive Disorder eFigure 3. Cumulative Incidence Function of LTC in Bipolar Disorder eFigure 4. Cumulative Incidence Function of LTC in Major Depressive Disorder [file jamanetwopen-e2020213-s001.pdf]

# Supplemental Online Content

Frey BN, Vigod S, de Azevedo Cardoso T, et al. The early burden of disability in individuals with mood and other common mental disorders in Ontario, Canada. *JAMA Netw Open*. 2020;3(10):e2020213.
doi:10.1001/jamanetworkopen.2020.20213

**eTable 1.** Diagnosis Codes
**eTable 2.** Selection of Cohorts
**eTable 3.** Complete Sociodemographic Information for Cohorts of Individuals With Mood Disorders, Common Mental Disorders, and Matched Individuals
**eTable 4.** Algorithms Used to Identify Chronic Conditions at Time of Mood Disorder Diagnosis
**eTable 5.** Crude Rates of Disability for the Mood Disorder Cohort
**eTable 6.** Crude Rates of Disability for the Common Mental Disorder Cohort
**eTable 7.** Baseline Sociodemographic Information for Individuals with Major Depressive Disorders, Bipolar Disorders, and Matched Individuals
**eTable 8.** Crude Rates of Disability for Bipolar Disorder
**eTable 9.** Crude Rates of Disability for Major Depressive Disorder
**eTable 10.** Crude Rate Ratio of Disability for Bipolar Disorder
**eTable 11.** Crude Rate Ratio of Disability for Major Depressive Disorder
**eTable 12.** Adjusted Hazard Ratios for Major Depressive and Bipolar Disorders
**eFigure 1.** Cumulative Incidence Function of ODSP in Bipolar Disorder
**eFigure 2.** Cumulative Incidence Function of ODSP in Major Depressive Disorder
**eFigure 3.** Cumulative Incidence Function of LTC in Bipolar Disorder
**eFigure 4.** Cumulative Incidence Function of LTC in Major Depressive Disorder

This supplemental material has been provided by the authors to give readers additional information about their work.

© 2020 Frey BN et al. *JAMA Network Open.*

**eTable 1. Diagnosis Codes**

**eTable 1a.** ICD-9 Mood Disorder Diagnosis Codes (Used for outpatient OHIP physician visits and hospitalizations from 1997-2002).

| ICD-9Code | Description | Excluded in specific cohort |
|---|---|---|
| 29600 | MANIC DISORDER-UNSPEC | |
| 29601 | MANIC DISORDER-MILD | |
| 29602 | MANIC DISORDER-MOD | |
| 29603 | MANIC DISORDER-SEVERE | |
| 29604 | MANIC DIS-SEVERE W PSYCH | |
| 29605 | MANIC DIS-PARTIAL REMISS | |
| 29606 | MANIC DIS-FULL REMISSION | |
| 29610 | RECUR MANIC DIS-UNSPEC | |
| 29611 | RECUR MANIC DIS-MILD | |
| 29612 | RECUR MANIC DIS-MOD | |
| 29613 | RECUR MANIC DIS-SEVERE | |
| 29614 | RECUR MANIC-SEV W PSYCHO | |
| 29615 | RECUR MANIC-PART REMISS | |
| 29616 | RECUR MANIC-FULL REMISS | |
| 29620 | DEPRESS PSYCHOSIS-UNSPEC | |
| 29621 | DEPRESS PSYCHOSIS-MILD | |
| 29622 | DEPRESSIVE PSYCHOSIS-MOD | |
| 29623 | DEPRESS PSYCHOSIS-SEVERE | |
| 29624 | DEPR PSYCHOS-SEV W PSYCH | |
| 29625 | DEPR PSYCHOS-PART REMISS | |
| 29626 | DEPR PSYCHOS-FULL REMISS | |
| 29630 | RECURR DEPR PSYCHOS-UNSP | |
| 29631 | RECURR DEPR PSYCHOS-MILD | |
| 29632 | RECURR DEPR PSYCHOS-MOD | |
| 29633 | RECUR DEPR PSYCH-SEVERE | |
| 29634 | REC DEPR PSYCH-PSYCHOTIC | |
| 29635 | RECUR DEPR PSYC-PART REM | |
| 29636 | RECUR DEPR PSYC-FULL REM | |
| 29640 | BIPOL AFF, MANIC-UNSPEC | |
| 29641 | BIPOLAR AFF, MANIC-MILD | |
| 29642 | BIPOLAR AFFEC, MANIC-MOD | |
| 29643 | BIPOL AFF, MANIC-SEVERE | |
| 29644 | BIPOL MANIC-SEV W PSYCH | |
| 29645 | BIPOL AFF MANIC-PART REM | |
| 29646 | BIPOL AFF MANIC-FULL REM | |
| 29650 | BIPOLAR AFF, DEPR-UNSPEC | |
| 29651 | BIPOLAR AFFEC, DEPR-MILD | |

© 2020 Frey BN et al. *JAMA Network Open.*

| | | |
|---|---|---|
| 29652 | BIPOLAR AFFEC, DEPR-MOD | |
| 29653 | BIPOL AFF, DEPR-SEVERE | |
| 29654 | BIPOL DEPR-SEV W PSYCH | |
| 29655 | BIPOL AFF DEPR-PART REM | |
| 29656 | BIPOL AFF DEPR-FULL REM | |
| 29660 | BIPOL AFF, MIXED-UNSPEC | |
| 29661 | BIPOLAR AFF, MIXED-MILD | |
| 29662 | BIPOLAR AFFEC, MIXED-MOD | |
| 29663 | BIPOL AFF, MIXED-SEVERE | |
| 29664 | BIPOL MIXED-SEV W PSYCH | |
| 29665 | BIPOL AFF, MIX-PART REM | |
| 29666 | BIPOL AFF, MIX-FULL REM | |
| 2967 | BIPOLAR AFFECTIVE NOS | |
| 29680 | MANIC-DEPRESSIVE NOS | |
| 29681 | ATYPICAL MANIC DISORDER | |
| 29682 | ATYPICAL DEPRESSIVE DIS | |
| 29689 | MANIC-DEPRESSIVE NEC | |
| 29690 | AFFECTIVE PSYCHOSIS NOS | |
| 29699 | AFFECTIVE PSYCHOSES NEC | |
| 30000 | ANXIETY STATE NOS | X |
| 30001 | PANIC DISORDER | X |
| 30002 | GENERALIZED ANXIETY DIS | X |
| 30009 | ANXIETY STATE NEC | X |
| 30010 | HYSTERIA NOS | X |
| 30011 | CONVERSION DISORDER | X |
| 30012 | PSYCHOGENIC AMNESIA | X |
| 30013 | PSYCHOGENIC FUGUE | X |
| 30014 | MULTIPLE PERSONALITY | X |
| 30015 | DISSOCIATIVE REACT NOS | X |
| 30016 | FACTITIOUS ILL W SYMPTOM | X |
| 30019 | FACTITIOUS ILL NEC/NOS | X |
| 30020 | PHOBIA NOS | X |
| 30021 | AGORAPHOBIA WITH PANIC | X |
| 30022 | AGORAPHOBIA W/O PANIC | X |
| 30023 | SOCIAL PHOBIA | X |
| 30029 | ISOLATED PHOBIAS NEC | X |
| 3003 | OBSESSIVE-COMPULSIVE DIS | X |
| 3004 | NEUROTIC DEPRESSION | X |
| 3005 | NEURASTHENIA | X |
| 3006 | DEPERSONALIZATION SYND | X |
| 3007 | HYPOCHONDRIASIS | X |
| 30081 | SOMATIZATION DISORDER | X |
| 30089 | NEUROTIC DISORDERS NEC | X |

© 2020 Frey BN et al. *JAMA Network Open.*

| | | |
|---|---|---|
| 3009 | NEUROTIC DISORDER NOS | X |
| 311 | DEPRESSIVE DISORDER NEC | |

**eTable 1b.** ICD-10 Mood Disorder Diagnosis Codes (Used for outpatient mood disorder hospitalizations from 2002-2007).

| ICD-10 Code | Description | Excluded in specific cohort |
|---|---|---|
| F30·0 | Hypomania | |
| F30·1 | Mania without psychotic symptoms | |
| F30·2 | Mania with psychotic symptoms | |
| F30·8 | Other manic episodes | |
| F30·9 | Manic episode, unspecified | |
| F31·0 | Bipolar affective disorder, current episode hypomanic | |
| F31·1 | Bipolar affective disorder, current episode manic without psychotic symptoms | |
| F31·2 | Bipolar affective disorder, current episode manic with psychotic symptoms | |
| F31·3 | Bipolar affective disorder, current episode mild or moderate depression | |
| F31·4 | Bipolar affective disorder, current episode severe depression without psychotic symptoms | |
| F31·5 | Bipolar affective disorder, current episode severe depression with psychotic symptoms | |
| F31·6 | Bipolar affective disorder, current episode mixed | |
| F31·7 | Bipolar affective disorder, currently in remission | |
| F31·8 | Other bipolar affective disorders | |
| F31·9 | Bipolar affective disorder, unspecified | |
| F32·0 | Mild depressive episode | |
| F32·1 | Moderate depressive episode | |
| F32·2 | Severe depressive episode without psychotic symptoms | |
| F32·3 | Severe depressive episode with psychotic symptoms | |
| F32·8 | Other depressive episodes | |
| F32·9 | Depressive episode, unspecified | |
| F33·0 | Recurrent depressive disorder, current episode mild | |
| F33·1 | Recurrent depressive disorder, current episode moderate | |
| F33·2 | Recurrent depressive disorder, current episode severe without psychotic symptoms | |
| F33·3 | Recurrent depressive disorder, current episode severe with psychotic symptoms | |

© 2020 Frey BN et al. *JAMA Network Open.*

| | | |
|---|---|---|
| F33·4 | Recurrent depressive disorder, currently in remission | |
| F33·8 | Other recurrent depressive disorders | |
| F33·9 | Recurrent depressive disorder, unspecified | |
| F34·0 | Cyclothymia | |
| F34·1 | Dysthymia | x |
| F34·8 | Other persistent mood [affective] disorders | |
| F34·9 | Persistent mood [affective] disorder, unspecified | |
| F38·0 | Other single mood [affective] disorders | |
| F38·1 | Other recurrent mood [affective] disorders | |
| F38·8 | Other specified mood [affective] disorders | |
| F39 | Unspecified mood [affective] disorder | |
| F40·0 | Agoraphobia | x |
| F40·1 | Social phobias | x |
| F40·2 | Specific (isolated) phobias | x |
| F40·8 | Other phobic anxiety disorders | x |
| F40·9 | Phobic anxiety disorder, unspecified | x |
| F41·0 | Panic disorder [episodic paroxysmal anxiety] | x |
| F41·1 | Generalized anxiety disorder | x |
| F41·2 | Mixed anxiety and depressive disorder | x |
| F41·3 | Other mixed anxiety disorders | x |
| F41·8 | Other specified anxiety disorders | x |
| F41·9 | Anxiety disorder, unspecified | x |
| F42·0 | Predominantly obsessional thoughts or ruminations | x |
| F42·1 | Predominantly compulsive acts [obsessional rituals] | x |
| F42·2 | Mixed obsessional thoughts and acts | x |
| F42·8 | Other obsessive-compulsive disorders | x |
| F42·9 | Obsessive-compulsive disorder, unspecified | x |
| F44·0 | Dissociative amnesia | x |
| F44·1 | Dissociative fugue | x |
| F44·2 | Dissociative stupor | x |
| F44·3 | Trance and possession disorders | x |
| F44·4 | Dissociative motor disorders | x |
| F44·5 | Dissociative convulsions | x |
| F44·6 | Dissociative anaesthesia and sensory loss | x |
| F44·7 | Mixed dissociative [conversion] disorders | x |
| F44·8 | Other dissociative [conversion] disorders | x |
| F44·9 | Dissociative [conversion] disorder, unspecified | x |
| F45·0 | Somatization disorder | x |
| F45·2 | Hypochondriacal disorder | x |
| F48·0 | Neurasthenia | x |

| | | |
|---|---|---|
| F48·1 | Depersonalization-derealization syndrome | x |
| F48·8 | Other specified neurotic disorders | x |
| F48·9 | Neurotic disorder, unspecified | x |
| F53·0 | Mild mental and behavioural disorders associated with the puerperium, not elsewhere classified | |
| F68·0 | Elaboration of physical symptoms for psychological reasons | x |

**eTable 1c.** DSM-IV Mood Disorder Diagnosis Codes (Used for inpatient hospitalizations from 2005-2007 in the OMHRS database).

| DSM-IV | Description | Excluded in specific cohort |
|---|---|---|
| 29600 | BIPOLAR I DISORDER, SINGLE MANIC EPISODE, UNSPECIFIED | |
| 29601 | BIPOLAR I DISORDER, SINGLE MANIC EPISODE, MILD | |
| 29602 | BIPOLAR I DISORDER, SINGLE MANIC EPISODE, MODERATE | |
| 29603 | BIPOLAR I DISORDER, SINGLE MANIC EPISODE, SEVERE WITHOUT PSYCHOTIC FEATURES | |
| 29604 | BIPOLAR I DISORDER, SINGLE MANIC EPISODE, SEVERE WITH PSYCHOTIC FEATURES | |
| 29605 | BIPOLAR I DISORDER, SINGLE MANIC EPISODE, IN PARTIAL REMISSION | |
| 29606 | BIPOLAR I DISORDER, SINGLE MANIC EPISODE, IN FULL REMISSION | |
| 29620 | MAJOR DEPRESSIVE DISORDER, SINGLE EPISODE, UNSPECIFIED | |
| 29621 | MAJOR DEPRESSIVE DISORDER, SINGLE EPISODE, MILD | |
| 29622 | MAJOR DEPRESSIVE DISORDER, SINGLE EPISODE, MODERATE | |
| 29623 | MAJOR DEPRESSIVE DISORDER, SINGLE EPISODE, SEVERE WITHOUT PSYCHOTIC FEATURES | |
| 29624 | MAJOR DEPRESSIVE DISORDER, SINGLE EPISODE, SEVERE WITH PSYCHOTIC FEATURES | |
| 29625 | MAJOR DEPRESSIVE DISORDER, SINGLE EPISODE, IN PARTIAL REMISSION | |
| 29626 | MAJOR DEPRESSIVE DISORDER, SINGLE EPISODE, IN FULL REMISSION | |

© 2020 Frey BN et al. *JAMA Network Open.*

| | |
|---|---|
| 29630 | MAJOR DEPRESSIVE DISORDER, RECURRENT, UNSPECIFIED |
| 29631 | MAJOR DEPRESSIVE DISORDER, RECURRENT, MILD |
| 29632 | MAJOR DEPRESSIVE DISORDER, RECURRENT, MODERATE |
| 29633 | MAJOR DEPRESSIVE DISORDER, RECURRENT, SEVERE WITHOUT PSYCHOTIC FEATURES |
| 29634 | MAJOR DEPRESSIVE DISORDER, RECURRENT, SEVERE WITH PSYCHOTIC FEATURES |
| 29635 | MAJOR DEPRESSIVE DISORDER, RECURRENT, IN PARTIAL REMISSION |
| 29636 | MAJOR DEPRESSIVE DISORDER, RECURRENT, IN FULL REMISSION |
| 29640 | BIPOLAR I DISORDER, MOST RECENT EPISODE MANIC, HYPO OR UNSPECIFIED |
| 29641 | BIPOLAR I DISORDER, MOST RECENT EPISODE MANIC, MILD |
| 29642 | BIPOLAR I DISORDER, MOST RECENT EPISODE MANIC, MODERATE |
| 29643 | BIPOLAR I DISORDER, MOST RECENT EPISODE MANIC, SEVERE WITHOUT PSYCHOTIC FEATURES |
| 29644 | BIPOLAR I DISORDER, MOST RECENT EPISODE MANIC, SEVERE WITH PSYCHOTIC FEATURES |
| 29645 | BIPOLAR I DISORDER, MOST RECENT EPISODE MANIC, IN PARTIAL REMISSION |
| 29646 | BIPOLAR I DISORDER, MOST RECENT EPISODE MANIC, IN FULL REMISSION |
| 29650 | BIPOLAR I DISORDER, MOST RECENT EPISODE DEPRESSED, UNSPECIFIED |
| 29651 | BIPOLAR I DISORDER, MOST RECENT EPISODE DEPRESSED, MILD |
| 29652 | BIPOLAR I DISORDER, MOST RECENT EPISODE DEPRESSED, MODERATE |
| 29653 | BIPOLAR I DISORDER, MOST RECENT EPISODE DEPRESSED, SEVERE WITHOUT PSYCHOTIC FEAT |
| 29654 | BIPOLAR I DISORDER, MOST RECENT EPISODE DEPRESSED, SEVERE WITH PSYCHOTIC FEATURE |

© 2020 Frey BN et al. *JAMA Network Open.*

| | | |
|---|---|---|
| 29655 | BIPOLAR I DISORDER, MOST RECENT EPISODE DEPRESSED, IN PARTIAL REMISSION | |
| 29656 | BIPOLAR I DISORDER, MOST RECENT EPISODE DEPRESSED, IN FULL REMISSION | |
| 29660 | BIPOLAR I DISORDER, MOST RECENT EPISODE MIXED, UNSPECIFIED | |
| 29661 | BIPOLAR I DISORDER, MOST RECENT EPISODE MIXED, MILD | |
| 29662 | BIPOLAR I DISORDER, MOST RECENT EPISODE MIXED, MODERATE | |
| 29663 | BIPOLAR I DISORDER, MOST RECENT EPISODE MIXED, SEVERE WITHOUT PSYCHOTIC FEATURES | |
| 29664 | BIPOLAR I DISORDER, MOST RECENT EPISODE MIXED, SEVERE WITH PSYCHOTIC FEATURES | |
| 29665 | BIPOLAR I DISORDER, MOST RECENT EPISODE MIXED, IN PARTIAL REMISSION | |
| 29666 | BIPOLAR I DISORDER, MOST RECENT EPISODE MIXED, IN FULL REMISSION | |
| 29670 | BIPOLAR I DISORDER, MOST RECENT EPISODE UNSPECIFIED | |
| 29680 | BIPOLAR DISORDER NOS | |
| 29689 | BIPOLAR II DISORDER | |
| 29690 | MOOD DISORDER NOS | |
| 30000 | ANXIETY DISORDER NOS | x |
| 30001 | PANIC DISORDER WITHOUT AGORAPHOBIA | x |
| 30002 | GENERALIZED ANXIETY DISORDER | x |
| 30011 | CONVERSION DISORDER | x |
| 30012 | DISSOCIATIVE AMNESIA | x |
| 30013 | DISSOCIATIVE FUGUE | x |
| 30014 | DISSOCIATIVE IDENTITY DISORDER | x |
| 30015 | DISSOCIATIVE DISORDER NOS | x |
| 30016 | FACTITIOUS DISORDER WITH PREDOMINANTLY PSYCHOLOGICAL SIGNS AND SYMPTOMS | x |
| 30019 | FACTITIOUS DISORDER NOS | x |
| 30021 | PANIC DISORDER WITH AGORAPHOBIA | x |
| 30022 | AGORAPHOBIA WITHOUT HISTORY OF PANIC DISORDER | x |
| 30023 | SOCIAL PHOBIA | x |
| 30029 | SPECIFIC PHOBIA | x |
| 30030 | OBSESSIVE-COMPULSIVE DISORDER | x |

© 2020 Frey BN et al. *JAMA Network Open.*

| | | |
|---|---|---|
| 30040 | DYSTHYMIC DISORDER | x |
| 30070 | SOMATIZATION DISORDER - BODY DYSMORPHIC OR HYPOCHONDRIASIS | x |
| 30081 | SOMATIZATION DISORDER | x |
| 30082 | SOMATOFORM DISORDER - UNDIFFERENTIATED AND NOS | x |
| 3009 | UNSPECIFIED MENTAL DISORDER (NONPSYCHOTIC) | x |
| 30113 | CYCLOTHYMIC DISORDER | |

© 2020 Frey BN et al. *JAMA Network Open.*

**eTable 1d:** Codes used to create the Major Depression group for the sensitivity analyses

| Source | Code |
|---|---|
| OHIP | 311 |
| ICD-10 | F32, F33, F38, F39 |
| ICD-9 | 29620 DEPRESS PSYCHOSIS-UNSPEC |
| ICD-9 | 29621 DEPRESS PSYCHOSIS-MILD |
| ICD-9 | 29622 DEPRESSIVE PSYCHOSIS-MOD |
| ICD-9 | 29623 DEPRESS PSYCHOSIS-SEVERE |
| ICD-9 | 29624 DEPR PSYCHOS-SEV W PSYCH |
| ICD-9 | 29625 DEPR PSYCHOS-PART REMISS |
| ICD-9 | 29626 DEPR PSYCHOS-FULL REMISS |
| ICD-9 | 29630 RECURR DEPR PSYCHOS-UNSP |
| ICD-9 | 29631 RECURR DEPR PSYCHOS-MILD |
| ICD-9 | 29632 RECURR DEPR PSYCHOS-MOD |
| ICD-9 | 29633 RECUR DEPR PSYCH-SEVERE |
| ICD-9 | 29634 REC DEPR PSYCH-PSYCHOTIC |
| ICD-9 | 29635 RECUR DEPR PSYC-PART REM |
| ICD-9 | 29690 AFFECTIVE PSYCHOSIS NOS |
| ICD-9 | 29699 AFFECTIVE PSYCHOSES NEC |
| ICD-9 | 29682 ATYPICAL DEPRESSIVE DIS |
| ICD-9 | 29699 AFFECTIVE PSYCHOSES NEC |
| ICD-9 | 29636 RECUR DEPR PSYC-FULL REM |
| ICD-9 | 29620 MAJOR DEPRESSIVE DISORDER, SINGLE EPISODE, UNSPECIFIED |
| ICD-9 | 29621 MAJOR DEPRESSIVE DISORDER, SINGLE EPISODE, MILD |
| ICD-9 | 29622 MAJOR DEPRESSIVE DISORDER, SINGLE EPISODE, MODERATE |
| ICD-9 | 29623 MAJOR DEPRESSIVE DISORDER, SINGLE EPISODE, SEVERE WITHOUT PSYCHOTIC FEATURES |
| ICD-9 | 29624 MAJOR DEPRESSIVE DISORDER, SINGLE EPISODE, SEVERE WITH PSYCHOTIC FEATURES |
| ICD-9 | 29625 MAJOR DEPRESSIVE DISORDER, SINGLE EPISODE, IN PARTIAL REMISSION |
| ICD-9 | 29626 MAJOR DEPRESSIVE DISORDER, SINGLE EPISODE, IN FULL REMISSION |
| ICD-9 | 29630 MAJOR DEPRESSIVE DISORDER, RECURRENT, UNSPECIFIED |
| ICD-9 | 29631 MAJOR DEPRESSIVE DISORDER, RECURRENT, MILD |
| ICD-9 | 29632 MAJOR DEPRESSIVE DISORDER, RECURRENT, MODERATE |
| ICD-9 | 29633 MAJOR DEPRESSIVE DISORDER, RECURRENT, SEVERE WITHOUT PSYCHOTIC FEATURES |
| ICD-9 | 29634 MAJOR DEPRESSIVE DISORDER, RECURRENT, SEVERE WITH PSYCHOTIC FEATURES |

© 2020 Frey BN et al. *JAMA Network Open.*

| | |
|---|---|
| ICD-9 | 29635 MAJOR DEPRESSIVE DISORDER, RECURRENT, IN PARTIAL REMISSION |
| | 311 DEPRESSIVE DISORDER NEC |
| ICD-9 | 29636 MAJOR DEPRESSIVE DISORDER, RECURRENT, IN FULL REMISSION |
| ICD-9 | 29690 MOOD DISORDER NOS |

**Table S1e:** Codes used to create the Bipolar Disorder group for the sensitivity analyses

| Source | Code |
|---|---|
| OHIP | 296 |
| ICD-10 | F30, F31, F34.0 |
| ICD-9 | 29600 MANIC DISORDER-UNSPEC |
| ICD-9 | 29601 MANIC DISORDER-MILD |
| ICD-9 | 29602 MANIC DISORDER-MOD |
| ICD-9 | 29603 MANIC DISORDER-SEVERE |
| ICD-9 | 29604 MANIC DIS-SEVERE W PSYCH |
| ICD-9 | 29605 MANIC DIS-PARTIAL REMISS |
| ICD-9 | 29606 MANIC DIS-FULL REMISSION |
| ICD-9 | 29610 RECUR MANIC DIS-UNSPEC |
| ICD-9 | 29611 RECUR MANIC DIS-MILD |
| ICD-9 | 29612 RECUR MANIC DIS-MOD |
| ICD-9 | 29613 RECUR MANIC DIS-SEVERE |
| ICD-9 | 29614 RECUR MANIC-SEV W PSYCHO |
| ICD-9 | 29615 RECUR MANIC-PART REMISS |
| ICD-9 | 29616 RECUR MANIC-FULL REMISS |
| ICD-9 | 29640 BIPOL AFF, MANIC-UNSPEC |
| ICD-9 | 29641 BIPOLAR AFF, MANIC-MILD |
| ICD-9 | 29642 BIPOLAR AFFEC, MANIC-MOD |
| ICD-9 | 29643 BIPOL AFF, MANIC-SEVERE |
| ICD-9 | 29644 BIPOL MANIC-SEV W PSYCH |
| ICD-9 | 29645 BIPOL AFF MANIC-PART REM |
| ICD-9 | 29646 BIPOL AFF MANIC-FULL REM |
| ICD-9 | 29650 BIPOLAR AFF, DEPR-UNSPEC |
| ICD-9 | 29651 BIPOLAR AFFEC, DEPR-MILD |
| ICD-9 | 29652 BIPOLAR AFFEC, DEPR-MOD |
| ICD-9 | 29653 BIPOL AFF, DEPR-SEVERE |
| ICD-9 | 29600 BIPOLAR I DISORDER, SINGLE MANIC EPISODE, UNSPECIFIED |
| ICD-9 | 29601 BIPOLAR I DISORDER, SINGLE MANIC EPISODE, MILD |
| ICD-9 | 29602 BIPOLAR I DISORDER, SINGLE MANIC EPISODE, MODERATE |
| ICD-9 | 29603 BIPOLAR I DISORDER, SINGLE MANIC EPISODE, SEVERE WITHOUT PSYCHOTIC FEATURES |

© 2020 Frey BN et al. *JAMA Network Open.*

| | |
|---|---|
| ICD-9 | 29604 BIPOLAR I DISORDER, SINGLE MANIC EPISODE, SEVERE WITH PSYCHOTIC FEATURES |
| ICD-9 | 29605 BIPOLAR I DISORDER, SINGLE MANIC EPISODE, IN PARTIAL REMISSION |
| ICD-9 | 29606 BIPOLAR I DISORDER, SINGLE MANIC EPISODE, IN FULL REMISSION |

© 2020 Frey BN et al. *JAMA Network Open.*

## eTable 2. Selection of Cohorts

**eTable 2a.** Selection of the specific mood disorder cohort.

| Step | Criteria | Description | n | % |
|---|---|---|---|---|
| 1 | Inclusion | Incident mood disorder diagnoses with discharge between Oct 1 1997 - March 31 2007 | 247 540 | |
| 2 | Exclusion | Non linkage (Death Date prior to index) | 247 455 | 99·97 |
| 3 | Exclusion | Out of Province Residents and no health care contact 7 years prior to index event | 246 669 | 99·65 |
| 4 | Exclusion | Ineligible for OHIP at Index Date or 5 years prior to index | 210 051 | 85·16 |
| 5 | Exclusion | Resident of LTC home 6 months prior to index date | 206 296 | 98·21 |
| 6 | Exclusion | ODSP prior to cohort entry (6 months prior to index) | 201 818 | 97·83 |
| 7 | Exclusion | Less than 18 years of age | 139 148 | 68·95 |
| | | **Study Cohort** | 139 148 | |

© 2020 Frey BN et al. *JAMA Network Open.*

**eTable 2b.** Selection of the common mental disorders cohort.

| Step | Criteria | Description | n | % |
|---|---|---|---|---|
| 1 | Inclusion | Incident common mental disorder diagnoses with discharge between Oct 1 1997 - March 31 2007 | 1 393 705 | |
| 2 | Exclusion | Non linkage (Death Date prior to index) | 1 393 495 | 99·98 |
| 3 | Exclusion | Out of Province Residents and No health care contact 7 years prior to index event | 1 389 942 | 99·73 |
| 4 | Exclusion | Ineligible for OHIP at Index Date or 2 years prior to index | 1 172 746 | 84·37 |
| 5 | Exclusion | Resident of LTC home 6 months prior to index date | 1 157 866 | 98·73 |
| 6 | Exclusion | ODSP prior to cohort entry (6 months prior to index) | 1 135 581 | 98·08 |
| 7 | Exclusion | Less than 18 years of age and over 60 | 812 248 | 71·53 |
| | | **Study Cohort** | 812 248 | |

© 2020 Frey BN et al. *JAMA Network Open.*

**eTable 3.** Complete Sociodemographic Information for Cohorts of Individuals With Mood Disorders, Common Mental Disorders, and Matched Individuals

| | Mood disorder patients (N=139148) | Mood disorder controls (N=139148) | Common mental disorders (N=812248) | Common mental disorders controls (N=812248) |
|---|---|---|---|---|
| **Income quintile** | | | | |
| 1 (lowest) | 25869 (18·59) | 25147 (18·07) | 147277 (18·13) | 148113 (18·23) |
| 2 | 27329 (19·64) | 27415 (19·70) | 160616 (19·77) | 159958 (19·69) |
| 3 | 28592 (20·55) | 28029 (20·14) | 165428 (20·37) | 164045 (20·20) |
| 4 | 28376 (20·39) | 28945 (20·80) | 168160 (20·70) | 167296 (20·60) |
| 5 (highest) | 28330 (20·36) | 28859 (20·74) | 167466 (20·62) | 168523 (20·75) |
| Missing | 652 (0·47) | 753 (0·54) | 33 01 (0·41) | 4313 (0·53) |
| **RIO** | | | | |
| Urban | 91766 (65·95) | 97027 (69·73) | 573651 (70·63) | 565418 (69·61) |
| Suburban | 33555 (24·11) | 28603 (20·56) | 168064 (20·69) | 167314 (20·6) |
| Rural | 12160 (8·74) | 11715 (8·42) | 62820 (7·73) | 68580 (8·44) |
| Missing | 1667 (1·20) | 1803 (1·30) | 7713 (0·95) | 10936 (1·35) |
| **Urban/Rural** | | | | |
| Urban | 118746 (85·34) | 120141 (86·34) | 709114 (87·30) | 700279 (86·21) |
| Rural | 20222 (14·53) | 18738 (13·47) | 101857 (12·54) | 110424 (13·59) |
| Missing | 180 (0·13) | 269 (0·19) | 1277 (0·16) | 1545 (0·19) |
| **Community size** | | | | |
| 1.500.000+ | 46761 (33·61) | 56019 (40·26) | 324691 (39·97) | 326813 (40·24) |
| 500.000-1.499.000 | 20485 (14·72) | 17119 (12·30) | 104295 (12·84) | 99631 (12·27) |
| 100.000-499.999 | 35850 (25·76) | 34299 (24·65) | 204964 (25·23) | 199865 (24·61) |
| 10.000-99.999 | 15664 (11·26) | 12673 (9·11) | 75011 (9·23) | 73948 (9·1) |
| <10.000 | 19564 (14·06) | 18172 (13·06) | 99098 (12·2) | 106786 (13·15) |
| Missing | 824 (0·59) | 866 (0·62) | 4189 (0·52) | 5205 (0·64) |

© 2020 Frey BN et al. *JAMA Network Open.*

| | | | | |
|---|---|---|---|---|
| **Ontario Marginalization Index Material Deprivation** | | | | |
| 1 (least marginalized) | 27855 (20·02) | 28024 (20·14) | 166391 (20·49) | 163751 (20·16) |
| 2 | 28009 (20·13) | 28082 (20·18) | 163916 (20·18) | 163616 (20·14) |
| 3 | 27226 (19·57) | 27723 (19·92) | 158560 (19·52) | 160477 (19·76) |
| 4 | 26479 (19·03) | 26457 (19·01) | 154491 (19·02) | 155296 (19·12) |
| 5 (most marginalized) | 26330 (18·92) | 25634 (18·42) | 151351 (18·63) | 149259 (18·38) |
| Missing | 3249 (2·33) | 3228 (2·32) | 17539 (2·16) | 19849 (2·44) |
| **Residential instability** | | | | |
| 1 (least marginalized) | 26242 (18·86) | 29399 (21·13) | 162909 (20·06) | 170903 (21·04) |
| 2 | 28.709 (20·63) | 28997 (20·84) | 167045 (20·57) | 168190 (20·71) |
| 3 | 26932 (19·35) | 26350 (18·94) | 156069 (19·21) | 154517 (19·02) |
| 4 | 28250 (20·30) | 26650 (19·15) | 159709 (19·66) | 155754 (19·18) |
| 5 (most marginalized) | 25712 (18·48) | 24524 (17·62) | 148977 (18·34) | 143035 (17·61) |
| Missing | 3303 (2·37) | 3228 (2·32) | 17539 (2·16) | 19849 (2·44) |
| **Dependency** | | | | |
| 1 (least marginalized) | 29952 (21·53) | 30488 (21·91) | 181919 (22·40) | 176177 (21·69) |
| 2 | 28952 (20·81) | 29983 (21·55) | 171762 (21·15) | 173738 (21·39) |
| 3 | 28198 (20·26) | 27981 (20·11) | 162576 (20·02) | 165350 (20·36) |
| 4 | 26322 (18·92) | 25831 (18·56) | 149731 (18·43) | 149299 (18·38) |
| 5 (most marginalized) | 22421 (16·11) | 21637 (15·55) | 128721 (15·85) | 127835 (15·74) |
| Missing | 3303 (2·37) | 3228 (2·32) | 17539 (2·16) | 19849 (2·44) |
| **Ethnic Concentration** | | | | |
| 1 (least marginalized) | 25559 (18·37) | 23898 (17·17) | 138373 (17·04) | 140725 (17·33) |

© 2020 Frey BN et al. *JAMA Network Open.*

| | | | | |
|---|---|---|---|---|
| 2 | 29125 (20·93) | 26542 (19·07) | 158094 (19·46) | 156542 (19·27) |
| 3 | 28257 (20·31) | 26382 (18·96) | 160471 (19·76) | 154199 (18·98) |
| 4 | 27032 (19·43) | 27415 (19·70) | 166417 (20·49) | 157801 (19·43) |
| 5 (most marginalized) | 25872 (18·59) | 31683 (22·77) | 171354 (21·1) | 183132 (22·55) |
| Missing | 3303 (2·37) | 3228 (2·32) | 17539 (2·16) | 19849 (2·44) |
| **Charlson Comorbidity Index** | | | | |
| M (SD) | 0·23 (0·85) | 0·14 (0·60) | 0·19 (0·75) | 0·14 (0·62) |
| 0 | 49803 (35·79) | 38 163 (27·43) | 286629 (35·29) | 221672 (27·29) |
| 1 | 3127 (2·25) | 1817 (1·31) | 17085 (2·1) | 10.881 (1·34) |
| 2+ | 3106 (2·23) | 1494 (1·07) | 14592 (1·8) | 8563 (1·05) |
| Non-hospitalized | 83112 (59·73) | 97674 (70·19) | 493942 (60·81) | 571132 (70·31) |
| **Chronic Conditions** | | | | |
| M(SD) | 0·31 (0·59) | 0·23 (0·51) | 0·29 (0·57) | 0·22 (0·51) |
| Hypertension | 15458 (11·11) | 12212 (8·78) | 88944 (10·95) | 70131 (8·63) |
| Diabetes | 5900 (4·24) | 4512 (3·24) | 30132 (3·71) | 25846 (3·18) |
| Congestive Heart Failure | 493 (0·35) | 296 (0·21) | 2314 (0·28) | 1682 (0·21) |
| Acute Myocardial Infarction | 847 (0·61) | 472 (0·34) | 4268 (0·53) | 2633 (0·32) |
| COPD | 4011 (2·88) | 2683 (1·93) | 22178 (2·73) | 15304 (1·88) |
| Asthma | 15898 (11·43) | 11287 (8·11) | 90828 (11·18) | 63682 (7·84) |
| HIV | 285 (0·20) | 80 (0·06) | 859 (0·11) | 481 (0·06) |

© 2020 Frey BN et al. *JAMA Network Open.*

**eTable 4.** Algorithms Used to Identify Chronic Conditions at Time of Mood Disorder Diagnosis

| Algorithms for Chronic Conditions assessed at time of incident diagnosis | | | | |
|---|---|---|---|---|
| **Condition** | **Data Sources** | **Definition** | **Codes** | **Reference** |
| **Hypertension** | HYPER | Adults (20+):<br>≥1 Hosp or ≥2 OHIP in a two-year period. OR * 1 OHIP followed by OHIP/Hosp within two | | Tu K, Chen Z, Lipscombe LL, Canadian Hypertension Education Program Outcomes Research Taskforce. Prevalence and incidence of hypertension from 1995 to 2005: a population-based study. Canadian Medical Association Journal. 2008 May 20;178(11):1429-35. |
| **Diabetes** | ODD | Adults (19+):<br>≥2 OHIP dx in a two-year period<br>OR (starting in 2011)<br>≥1 Hosp or ≥1 OHIP fee-code (in a two-year period | OHIP:<br>dx 250<br>fee Q040 K029 K030<br>DAD dx 250 | Guttmann A, Nakhla M, Henderson M, To T, Daneman D, Cauch-Dudek K, Wang X, Lam K, Hux J. Validation of a health administrative data algorithm for assessing the epidemiology of diabetes in Canadian children. Pediatric diabetes. 2010 Mar 1;11(2):122-8. |
| **Congestive Heart Failure** | Ontario CHF Database | Adults (40+):<br>≥1 Hosp (DAD, SDS, OMHRS) or ≥1 OHIP/ED, followed by ≥1 Hosp/ED/OHIP within one year | | Schultz SE, Rothwell DM, Chen Z, Tu K. Identifying cases of congestive heart failure from administrative data: a validation study using primary care patient records. Chronic |

© 2020 Frey BN et al. *JAMA Network Open.*

| | | | | |
|---|---|---|---|---|
| | | | | diseases and injuries in Canada 2013;33:160-6. |
| **COPD** | COPD | Sensitive cohort (35+): ≥1 Hosp or ≥1 OHIP | | Gershon A, Wang C, Guan J, Vasilevska-Ristovska J, Cicutto L, To T. Identifying individuals with physcian diagnosed COPD in health administrative databases. COPD 2009;6:388-94. |
| **Asthma** | ASTHMA | Sensitive cohort (18+): ≥1 Hosp or ≥2 OHIP in a two-year period | | Gershon AS, Wang C, Guan J, Vasilevska-Ristovska J, Cicutto L, To T. Identifying patients with physician-diagnosed asthma in health administrative databases. Can Respir J 2009;16:183-8. * Andrea S. Gershon, Jun Guan, Chengning Wang, Teresa To; Trends in Asthma Prevalence and Incidence in Ontario, Canada, 1996–2005: A Population Study. Am J Epidemiol 2010; 172 (6): 728-736. doi: 10.1093/aje/kwq189 * To T, Dell S, Dick P, et al. Defining asthma in children for surveillance, Am J Respir Crit Care Med, 2004, vol. 169 7pg. A383 |
| **Acute Myocardial Infarctions (Previous Heart Attack)*** | OMID | Adults (20+):<br><br>≥1 Hosp with most responsible dx of AMI and no AMI Hosp in the previous year | | Austin PC, Daly PA, Tu JV. A multicenter study of the coding accuracy of hospital discharge administrative data for patients admitted to cardiac care units in Ontario. American heart journal 2002;144:290-6. |

© 2020 Frey BN et al. *JAMA Network Open.*

| | | | | |
|---|---|---|---|---|
| **HIV *** | HIV | Adults (18+): ≥3 OHIP in a three-year period | OHIP dxcode: 042, 043, 044<br><br>3 OHIP physician billings for HIV within 3 years<br><br>Index date= OHIP claim or previous hospitalization with HIV dxcode<br><br>ICD-9: 042, 043, 044,<br><br>ICD-10: B20, B21, B22, B23, B24,<br><br>OHIP dx code: 042, 043, 044 | Tony Antoniou, Brandon Zagorski, Mona R. Loutfy, Carol Strike, Richard H. Glazier. Validation of Case-Finding Algorithms Derived from Administrative Data for Identifying Adults Living with Human Immunodeficiency Virus Infection. Plos One. 2011;6(6):e21748. Epub 2011 Jun 30. |

© 2020 Frey BN et al. *JAMA Network Open.*

**eTable 5.** Crude Rates of Disability for the Mood Disorder Cohort

| | ODSP | | | LTC | | |
|---|---|---|---|---|---|---|
| | N (%) | Total person years | Crude rate of disability* | N (%) | Total person years | Crude rate of disability* |
| | | | **Mood disorders cohort (n = 139148)** | | | |
| Overall | 8888 (6·39) | 1725978 | 51·50 | 463 (0·33) | 1789866 | 2·59 |
| Female, 18 to 30 | 1502 (5·63) | 352194 | 42·65 | 11 (0·04) | 363535 | 0·30 |
| Female, 31 to 40 | 1129 (5·69) | 271773 | 41·54 | 25 (0·13) | 280260 | 0·89 |
| Female, 41 to 50 | 1099 (5·79) | 252067 | 43·60 | 89 (0·47) | 259918 | 3·42 |
| Female, 51 to 60 | 595 (4·51) | 113618 | 52·37 | 89 (0·68) | 116757 | 7·62 |
| Male, 18 to 30 | 2092 (9·08) | 292946 | 71·41 | 13 (0·05) | 324839 | 0·40 |
| Male, 31 to 40 | 1168 (7·47) | 210418 | 55·51 | 29 (0·19) | 219491 | 1·32 |
| Male, 41 to 50 | 1196 (7·71) | 200293 | 59·71 | 111 (0·72) | 208716 | 5·32 |
| Male, 51 to 60 | 576 (5·20) | 93078 | 61·88 | 98 (0·88) | 95858 | 10·22 |
| | | | **Population-based controls (n = 139148)** | | | |
| Overall | 3104 (2·23) | 1216316 | 25·52 | 123 (0·09) | 1235633 | 0·995 |
| Female, 18 to 30 | 391 (4·52) | 316414 | 38·18 | 12 (0·04) | 325360 | 0·37 |
| Female, 31 to 40 | 391 (1·97) | 175029 | 22·34 | 11 (0·06) | 177831 | 0·62 |
| Female, 41 to 50 | 456 (2·40) | 170393 | 26·76 | 24 (0·13) | 173387 | 1·38 |
| Female, 51 to 60 | 427 (3·24) | 86665 | 49·27 | 34 (0·26) | 88805 | 3·83 |
| Male, 18 to 30 | 387 (2·12) | 223842 | 17·29 | 10 (0·04) | 307691 | 0·33 |
| Male, 31 to 40 | 339 (2·17) | 156017 | 21·73 | 7 (0·04) | 158328 | 0·44 |
| Male, 41 to 50 | 372 (2·39) | 152583 | 24·38 | 17 (0·11) | 154825 | 1·10 |
| Male, 51 to 60 | 372 (3·37) | 76632 | 48·54 | 21 (0·19) | 78268 | 2·68 |

* per 10000 patients

© 2020 Frey BN et al. *JAMA Network Open.*

**eTable 6.** Crude Rates of Disability for the Common Mental Disorder Cohort

| | ODSP | | | LTC | | |
|---|---|---|---|---|---|---|
| | N (%) | Total person years | Crude rate of disability* | N (%) | Total person years | Crude rate of disability* |
| | **Common mental disorders cohort (n = 812248)** | | | | | |
| Overall | 46720 (5·75) | 10371421 | 45·05 | 2392 (0·29) | 10712492 | 2·23 |
| Female, 18 to 30 | 3532 (5·88) | 796175 | 44·36 | 26 (0·04) | 823603 | 0·32 |
| Female, 31 to 40 | 6193 (5·12) | 1714173 | 36·13 | 154 (0·13) | 1762839 | 0·87 |
| Female, 41 to 50 | 5886 (5·16) | 1561010 | 37·71 | 476 (0·42) | 1604879 | 2·97 |
| Female, 51 to 60 | 2937 (3·73) | 690512 | 42·53 | 464 (0·59) | 705901 | 6·57 |
| Male, 18 to 30 | 3990 (9·20) | 553394 | 72·10 | 47 (0·11) | 600863 | 0·78 |
| Male, 31 to 40 | 6520 (7·09) | 1269614 | 51·35 | 190 (0·21) | 1318783 | 1·44 |
| Male, 41 to 50 | 6005 (6·76) | 1181388 | 50·83 | 463 (0·52) | 1223375 | 3·78 |
| Male, 51 to 60 | 2866 (4·55) | 539122 | 53·16 | 463 (0·73) | 552960 | 8·37 |
| | **Population-based controls (n = 812248)** | | | | | |
| Overall | 28395 (3·49) | 10297928 | 27·57 | 1798 (0·22) | 10531582 | 1·71 |
| Female, 18 to 30 | 3244 (2·91) | 1494909 | 21·70 | 23 (0·04) | 814088 | 0·28 |
| Female, 31 to 40 | 4001 (3·31) | 1663057 | 24·06 | 148 (0·12) | 1703005 | 0·87 |
| Female, 41 to 50 | 4406 (3·87) | 1539674 | 28·62 | 407 (0·36) | 1577632 | 2·58 |
| Female, 51 to 60 | 3153 (4·00) | 687672 | 45·85 | 357 (0·45) | 707794 | 5·04 |
| Male, 18 to 30 | 1072 (2·78) | 508953 | 21·06 | 15 (0·02) | 598595 | 0·25 |
| Male, 31 to 40 | 3159 (3·43) | 1271580 | 24·84 | 128 (0·14) | 1296857 | 0·99 |
| Male, 41 to 50 | 3891 (4·38) | 1189974 | 32·70 | 316 (0·36) | 1218910 | 2·59 |
| Male, 51 to 60 | 2414 (3·84) | 545511 | 44·25 | 325 (0·52) | 558479 | 5·82 |

1 per 10000 patients

© 2020 Frey BN et al. *JAMA Network Open.*

**eTable 7.** Baseline Sociodemographic Information for Individuals with Major Depressive Disorders, Bipolar Disorders, and Matched Individuals

| | Major Depressive Disorders | | Bipolar Disorders | |
|---|---|---|---|---|
| | **Cases N=128,167** | **Controls N=128,167** | **Cases N=10,981** | **Controls N=10,981** |
| **Age (SD)** | 37.64 (11.92) | 37.64 (11.94) | 36.35 (12.09) | 36.35 (12.09) |
| **Female (N%)** | 73318 (57.21) | 73322 (57.21) | 5373 (48.93) | 5373 (48.93) |
| **Death (N%)** | 4391 (3.43) | 1702 (1.33) | 563 (5.13) | 145 (1.32) |
| **Type of incident event** | | | | |
| Inpatient | 515 (0.40) | N/A | 565 (5.15) | N/A |
| Outpatient | 127652 (99.60) | N/A | 10416 (94.85) | N/A |
| **Immigrant status** | | | | |
| <10 years | 5100 (3.98) | 7362 (5.74) | 467 (4.25) | 583 (5.31) |
| >10 years | 5471 (4.27) | 7756 (5.90) | 441 (4.02) | 610 (5.56) |
| Non-immigrant | 117596 (91.75) | 113249 (88.36) | 10073 (91.73) | 9788(89.14) |

© 2020 Frey BN et al. *JAMA Network Open.*

**eTable 8.** Crude Rates of Disability for Bipolar Disorder

| Characteristic | Age Group | N | % | Total Person Time | Crude Rate Disability | N | % | Total Person Time | Crude Rate Disability | Sex |
|---|---|---|---|---|---|---|---|---|---|---|
| **Overall-ODSP** | | 1500 | 13.66 | 129917,40 | 115.46 | 263 | 2.40 | 98168,24 | 26.79 | |
| **Age Groups-ODSP** | 18 to 20 | 80 | 11.68 | 8609,35 | 92.92 | 13 | 1.92 | 5663,16 | 22.96 | F |
| **Age Groups-ODSP** | 21 to 30 | 148 | 10.92 | 17432,91 | 84.90 | 29 | 2.12 | 11689,85 | 24.81 | F |
| **Age Groups-ODSP** | 31 to 40 | 147 | 11.34 | 17360,65 | 84.67 | 37 | 2.88 | 11543,25 | 32.05 | F |
| **Age Groups-ODSP** | 41 to 50 | 141 | 11.38 | 16094,92 | 87.61 | 32 | 2.56 | 11560,25 | 27.68 | F |
| **Age Groups-ODSP** | 51 to 60 | 91 | 11.40 | 6431,23 | 141.50 | 27 | 3.39 | 5292,90 | 51.01 | F |
| **Age Groups-ODSP** | 18 to 20 | 119 | 20.77 | 6473,65 | 183.82 | 6 | 1.06 | 5383,12 | 11.15 | M |
| **Age Groups-ODSP** | 21 to 30 | 255 | 18.02 | 16527,98 | 154.28 | 20 | 1.41 | 13469,75 | 14.85 | M |
| **Age Groups-ODSP** | 31 to 40 | 227 | 16.26 | 17630,95 | 128.75 | 41 | 2.95 | 14273,32 | 28.72 | M |
| **Age Groups-ODSP** | 41 to 50 | 210 | 15.56 | 16358,37 | 128.37 | 30 | 2.22 | 13328,49 | 22.51 | M |
| **Age Groups-ODSP** | 51 to 60 | 82 | 9.38 | 6997,40 | 117.19 | 28 | 3.20 | 5964,14 | 46.95 | M |
| **Overall-LTC** | | 82 | 0.75 | 141962,41 | 5.78 | 11 | 0.10 | 99910,06 | 1.10 | |
| **Age Groups-LTC** | 18 to 20 | NR | NR | 9314,52 | 0.00 | NR | NR | 5765,67 | 1.73 | F |
| **Age Groups-LTC** | 21 to 30 | NR | NR | 18670,22 | 0.54 | NR | NR | 11888,41 | 0.00 | F |
| **Age Groups-LTC** | 31 to 40 | NR | NR | 18538,73 | 1.62 | NR | NR | 11820,67 | 0.85 | F |
| **Age Groups-LTC** | 41 to 50 | 11 | 0.89 | 17216,23 | 6.39 | NR | NR | 11796,15 | 0.85 | F |
| **Age Groups-LTC** | 51 to 60 | 13 | 1.63 | 6909,90 | 18.81 | NR | NR | 5425,45 | 9.22 | F |
| **Age Groups-LTC** | 18 to 20 | NR | NR | 7604,53 | 1.32 | NR | NR | 5431,65 | 0.00 | M |
| **Age Groups-LTC** | 21 to 30 | 6 | 0.42 | 18658,49 | 3.22 | NR | NR | 13621,34 | 0.00 | M |
| **Age Groups-LTC** | 31 to 40 | 6 | 0.43 | 19555,33 | 3.07 | NR | NR | 14599,72 | 0.00 | M |
| **Age Groups-LTC** | 41 to 50 | 28 | 2.07 | 18044,64 | 15.52 | NR | NR | 13473,62 | 0.74 | M |
| **Age Groups-LTC** | 51 to 60 | 13 | 1.49 | 7449,81 | 17.45 | NR | NR | 6087,38 | 3.29 | M |

Cells with N<6 are not reportable (NR)

© 2020 Frey BN et al. *JAMA Network Open.*

**eTable 9.** Crude Rates of Disability for Major Depressive Disorder

| | Age Group | N | % | Total Person Time | Crude Rate of Disability | N | % | Total Person Time | Crude Rate of Disability | Sex |
|---|---|---|---|---|---|---|---|---|---|---|
| **Overall-ODSP** | | 7388 | 5.76 | 1596060,60 | 46.29 | 2841 | 2.22 | 1118147,90 | 25.41 | |
| **Age Groups-ODSP** | 18 to 20 | 367 | 5.27 | 90934,26 | 40.36 | 140 | 1.99 | 58100,61 | 24.10 | F |
| **Age Groups-ODSP** | 21 to 30 | 907 | 5.13 | 235217,11 | 38.56 | 274 | 1.56 | 146193,23 | 18.74 | F |
| **Age Groups-ODSP** | 31 to 40 | 982 | 5.29 | 254411,92 | 38.60 | 354 | 1.91 | 163485,30 | 21.65 | F |
| **Age Groups-ODSP** | 41 to 50 | 958 | 5.40 | 235971,79 | 40.60 | 424 | 2.39 | 158832,34 | 26.69 | F |
| **Age Groups-ODSP** | 51 to 60 | 504 | 4.07 | 107186,28 | 47.02 | 400 | 3.23 | 81372,11 | 49.16 | F |
| **Age Groups-ODSP** | 18 to 20 | 350 | 8.23 | 53933,40 | 64.89 | 90 | 2.12 | 41107,04 | 21.89 | M |
| **Age Groups-ODSP** | 21 to 30 | 899 | 7.50 | 155603,69 | 57.77 | 175 | 1.46 | 117391,96 | 14.91 | M |
| **Age Groups-ODSP** | 31 to 40 | 941 | 6.61 | 192786,92 | 48.81 | 298 | 2.09 | 141743,39 | 21.02 | M |
| **Age Groups-ODSP** | 41 to 50 | 986 | 6.96 | 183934,63 | 53.61 | 342 | 2.41 | 139254,39 | 24.56 | M |
| **Age Groups-ODSP** | 51 to 60 | 494 | 4.84 | 86080,60 | 57.39 | 344 | 3.38 | 70667,55 | 48.68 | M |
| **Overall-LTC** | | 381 | 0.30 | 1647903,76 | 2.31 | 112 | 0.09 | 1135722,78 | 0.99 | |
| **Age Groups-LTC** | 18 to 20 | NR | NR | 93651,08 | 0.11 | NR | NR | 59025,15 | 0.17 | F |
| **Age Groups-LTC** | 21 to 30 | 9 | 0.05 | 241898,76 | 0.37 | NR | NR | 148191,60 | 0.27 | F |
| **Age Groups-LTC** | 31 to 40 | 22 | 0.12 | 261721,20 | 0.84 | 10 | 0.05 | 166010,39 | 0.60 | F |
| **Age Groups-LTC** | 41 to 50 | 78 | 0.44 | 242701,66 | 3.21 | 23 | 0.13 | 161590,48 | 1.42 | F |
| **Age Groups-LTC** | 51 to 60 | 76 | 0.61 | 109847,16 | 6.92 | 29 | 0.23 | 83379,39 | 3.48 | F |
| **Age Groups-LTC** | 18 to 20 | NR | NR | 56665,03 | 0.35 | NR | NR | 41690,00 | 0.00 | M |
| **Age Groups-LTC** | 21 to 30 | NR | NR | 162403,72 | 0.12 | NR | NR | 118575,56 | 0.25 | M |
| **Age Groups-LTC** | 31 to 40 | 23 | 0.16 | 199935,74 | 1.15 | 7 | 0.05 | 143727,77 | 0.49 | M |
| **Age Groups-LTC** | 41 to 50 | 83 | 0.59 | 190671,08 | 4.35 | 16 | 0.11 | 141351,49 | 1.13 | M |
| **Age Groups-LTC** | 51 to 60 | 85 | 0.83 | 88408,33 | 9.61 | 19 | 0.19 | 72180,94 | 2.63 | M |

Cells with N<6 are not reportable (NR)

© 2020 Frey BN et al. *JAMA Network Open.*

**eTable 10.** Crude Rate Ratio of Disability for Bipolar Disorder

| | ODSP | | LTC | |
|---|---|---|---|---|
| | **Crude Rate Ratio** | **95 % CI** | **Crude Rate Ratio** | **95 % CI** |
| | | **Bipolar Disorder (N=10981)** | | |
| **Overall** | 4.31 | 3.56-5.17 | 5.25 | 1.88-11.58 |
| **Female, 18 to 20** | 4.05 | 3.27-4.96 | N/E | N/E |
| **Female, 21 to 30** | 3.42 | 2.73-4.23 | N/E | N/E |
| **Female, 31 to 40** | 2.64 | 2.11-3.77 | 1.91 | 0.16-7.81 |
| **Female, 41 to 50** | 3.16 | 2.54-3.90 | 7.54 | 2.87-16.0 |
| **Female, 51 to 60** | 2.77 | 2.33-3.27 | 2.04 | 1.23-3.19 |
| **Male, 18 to 20** | 16.49 | 14.2-19.1 | N/E | N/E |
| **Male, 21 to 30** | 10.39 | 8.82-12.2 | N/E | N/E |
| **Male, 31 to 40** | 4.48 | 3.74-5.33 | N/E | N/E |
| **Male, 41 to 50** | 5.70 | 4.76-6.78 | 20.91 | 11.8-34.2 |
| **Male, 51 to 60** | 2.50 | 2.06-2.99 | 5.31 | 3.12-8.45 |

© 2020 Frey BN et al. *JAMA Network Open.*

**eTable 11.** Crude Rate Ratio of Disability for Major Depressive Disorder

| | ODSP | | LTC | |
|---|---|---|---|---|
| | **Crude Rate Ratio** | **95 % CI** | **Crude Rate Ratio** | **95 % CI** |
| | **Major Depressive Disorder (N=128167)** | | | |
| **Overall** | 1.82 | 1.36-2.43 | 2.34 | 0.35-7.82 |
| **Female, 18 to 20** | 1.67 | 1.20-2.28 | 0.63 | 0.00-7.82 |
| **Female, 21 to 30** | 2.06 | 1.46-2.82 | 1.38 | 0.00-16.4 |
| **Female, 31 to 40** | 1.78 | 1.27-2.44 | 1.40 | 0.02-8.79 |
| **Female, 41 to 50** | 1.52 | 1.09-2.07 | 2.26 | 0.50-6.38 |
| **Female, 51 to 60** | 0.96 | 0.70-1.27 | 1.99 | 0.79-4.13 |
| **Male, 18 to 20** | 2.96 | 2.29-3.78 | N/E | N/E |
| **Male, 21 to 30** | 3.88 | 2.94-5.01 | 0.49 | 0.00-15.6 |
| **Male, 31 to 40** | 2.32 | 1.71-3.07 | 2.36 | 0.09-11.9 |
| **Male, 41 to 50** | 2.18 | 1.63-2.85 | 3.85 | 1.12-9.50 |
| **Male, 51 to 60** | 1.18 | 0.89-1.53 | 3.65 | 1.72-6.80 |

© 2020 Frey BN et al. *JAMA Network Open.*

**eTable 12.** Adjusted Hazard Ratios for Major Depressive and Bipolar Disorders

| | ODSP | | LTC | |
|---|---|---|---|---|
| | **HR** | **ProbChiSq** | **HR** | **ProbChiSq** |
| | **Bipolar Disorders** | | | |
| **Overall** | 4.31 | <0.0001 | 4.39 | <0.0001 |
| **Female, 18 to 30** | 3.48 | <0.0001 | NE | NE |
| **Female, 31 to 40** | 2.91 | <0.0001 | 0.91 | 0.94 |
| **Female, 41 to 50** | 3.13 | <0.0001 | 6.41 | 0.11 |
| **Female, 51 to 60** | 2.72 | <0.0001 | 2.28 | 0.92 |
| **Male, 18 to 30** | 11.5 | <0.0001 | >10 | <0.0001 |
| **Male, 31 to 40** | 4.49 | <0.0001 | >10 | <0.0001 |
| **Male 41 to 50** | 5.52 | <0.0001 | 14.3 | 0.009 |
| **Male 51 to 60** | 2.17 | 0.0008 | 4.51 | 0.05 |

| | ODSP | | LTC | |
|---|---|---|---|---|
| | **HR** | **ProbChiSq** | **HR** | **ProbChiSq** |
| | **Major Depressive Disorders** | | | |
| **Overall** | 1.83 | <0.0001 | 1.99 | <0.0001 |
| **Female, 18 to 30** | 1.85 | <0.0001 | 1.04 | 0.95 |
| **Female, 31 to 40** | 1.88 | <0.0001 | 1.17 | 0.69 |
| **Female, 41 to 50** | 1.57 | <0.0001 | 1.88 | 0.009 |
| **Female, 51 to 60** | 1 | 0.95 | 1.72 | 0.01 |
| **Male, 18 to 30** | 3.54 | <0.0001 | 0.86 | 0.86 |
| **Male, 31 to 40** | 2.29 | <0.0001 | 1.82 | 0.21 |
| **Male 41 to 50** | 2.08 | <0.0001 | 3.11 | <0.0001 |
| **Male 51 to 60** | 1.14 | 0.07 | 3.05 | <0.0001 |

© 2020 Frey BN et al. *JAMA Network Open.*

**eFigure 1.** Cumulative Incidence Function of ODSP in Bipolar Disorder

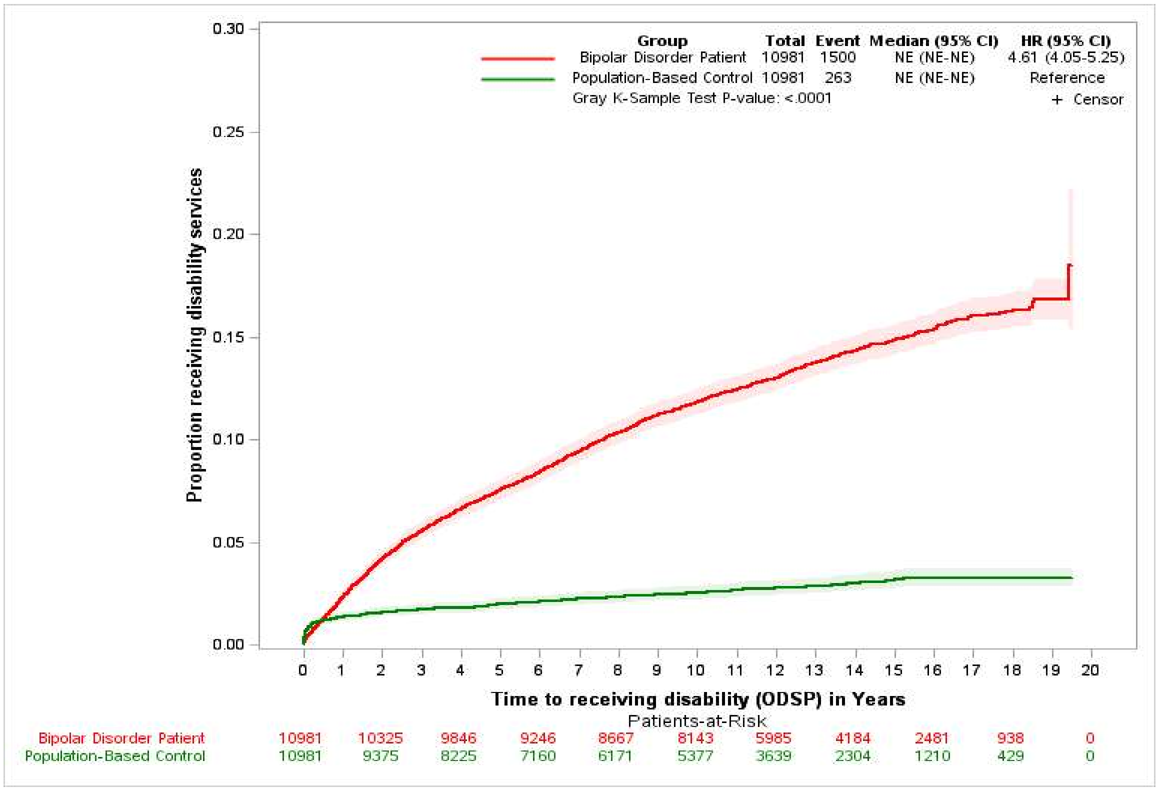


**Figure legend:** Bipolar disorder subjects compared to birth year and sex matched population-based controls (n=10981 per group). ODSP: Ontario Disability Support Program

© 2020 Frey BN et al. *JAMA Network Open.*

**eFigure 2.** Cumulative Incidence Function of ODSP in Major Depressive Disorder

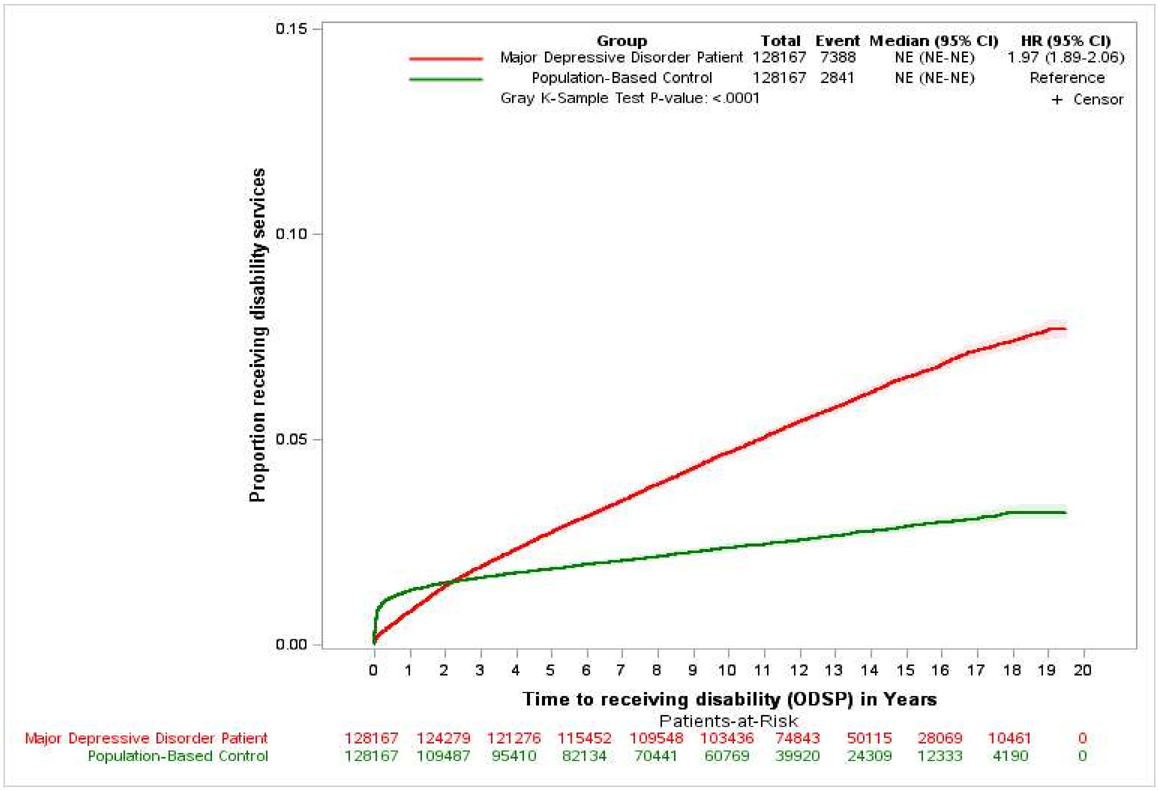


**Figure legend:** Major depressive disorder subjects compared to birth year and sex matched population-based controls (n=128167 per group). ODSP: Ontario Disability Support Program

© 2020 Frey BN et al. *JAMA Network Open.*

**eFigure 3.** Cumulative Incidence Function of LTC in Bipolar Disorder

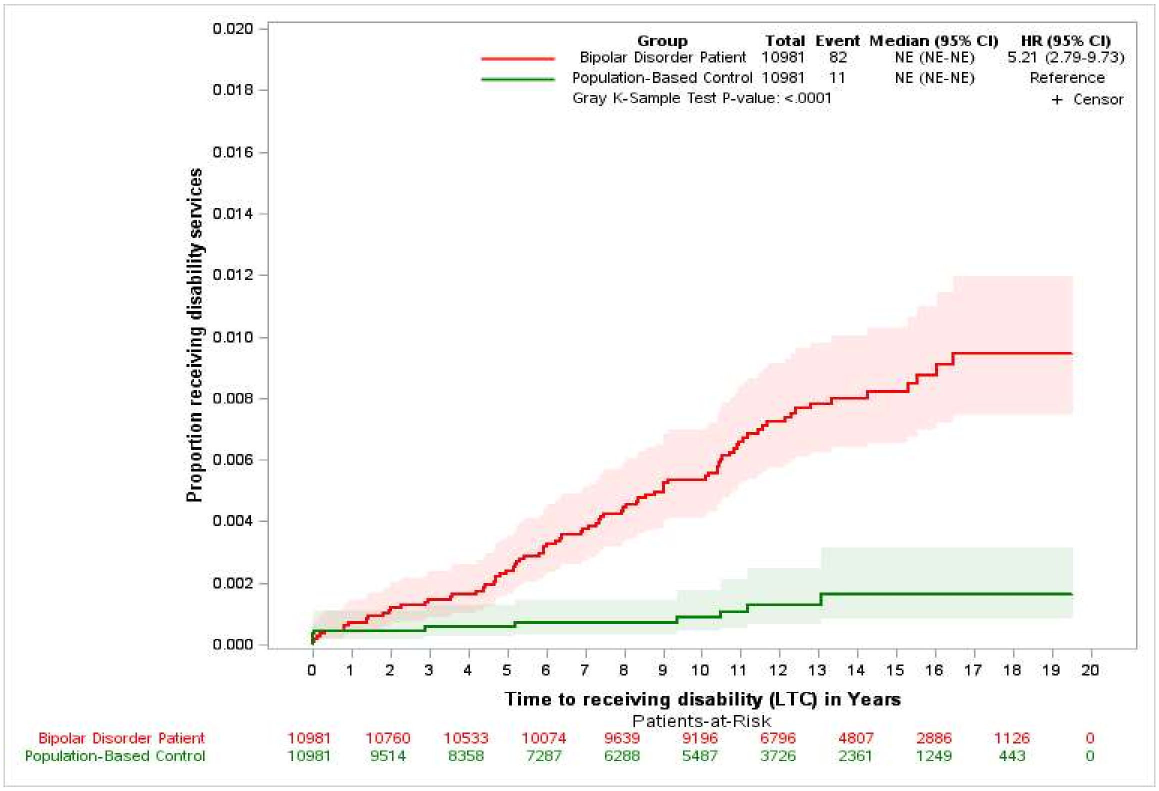


**Figure legend:** Bipolar disorder subjects compared to birth year and sex matched population-based controls (n=10981 per group). LTC: Long-term care

© 2020 Frey BN et al. *JAMA Network Open.*

**eFigure 4.** Cumulative Incidence Function of LTC in Major Depressive Disorder

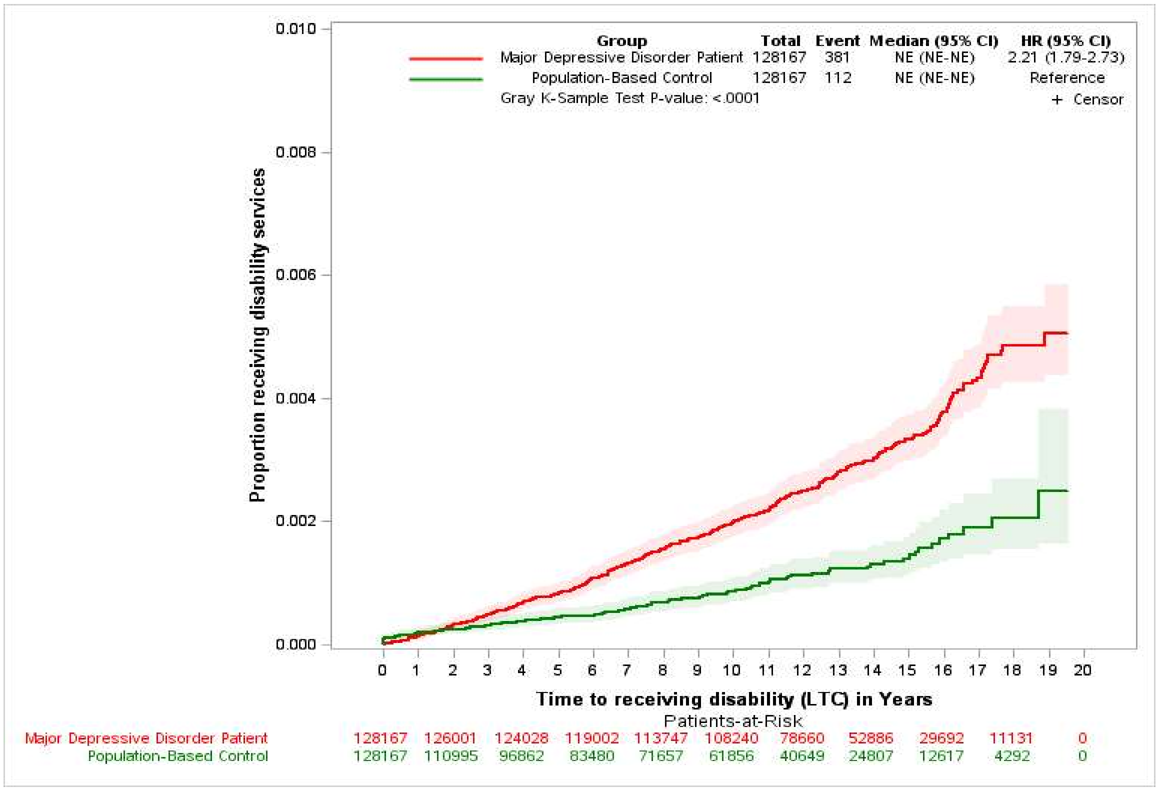


**Figure legend:** Major depressive disorder subjects compared to birth year and sex matched population-based controls (n=128167 per group). LTC: Long-term care

© 2020 Frey BN et al. *JAMA Network Open.*
